# A high-quality assembled genome of a representative peach landrace, ‘Feichenghongli’, and analysis of distinct late florescence and narrow leaf traits

**DOI:** 10.1186/s12870-023-04242-7

**Published:** 2023-04-29

**Authors:** Miao Li, Jian Li, Peixian Nie, Guixiang Li, Wei Liu, Qingtao Gong, Xiaomin Dong, Xiaolan Gao, Wenyu Chen, Anning Zhang

**Affiliations:** 1 Shandong Institute of Pomology, Taian City, 271000 Shandong Province China; 2 Feicheng peach Industry Development Center, Feicheng City, 271600 Shandong Province China

**Keywords:** Peach landraces, Genome assembly, Comparative genomics, Gene family, Late florescence, Narrow leaf

## Abstract

**Background:**

Peach (*Prunus persica* L. Batsch) is one of the most popular fruits worldwide. Although the reference genome of ‘Lovell’ peach has been released, the diversity of genome-level variations cannot be explored with one genome. To detect these variations, it is necessary to assemble more genomes.

**Results:**

We sequenced and *de novo* assembled the genome of ‘Feichenghongli’ (FCHL), a representative landrace with strict self-pollination, which maintained the homozygosity of the genome as much as possible. The chromosome-level genome of FCHL was 239.06 Mb in size with a contig N50 of 26.93 Mb and only 4 gaps at the scaffold level. The alignment of the FCHL genome with the reference ‘Lovell’ genome enabled the identification of 432535 SNPs, 101244 insertions and deletions, and 7299 structural variants. Gene family analysis showed that the expanded genes in FCHL were enriched in sesquiterpenoids and triterpenoid biosynthesis. RNA-seq analyses were carried out to investigate the two distinct traits of late florescence and narrow leaves. Two key genes, *PpDAM4* and *PpAGL31*, were identified candidates for the control of flower bud dormancy, and an F-box gene, *PpFBX92*, was identified as a good candidate gene in the regulation of leaf size.

**Conclusions:**

The assembled high-quality genome could deepen our understanding of variations among diverse genomes and provide valuable information for identifying functional genes and improving the molecular breeding process.

**Supplementary Information:**

The online version contains supplementary material available at 10.1186/s12870-023-04242-7.

## Background

Peach (*Prunus persica* L. Batsch), a species bearing delicious fruit, is one of the most popular fruit crops worldwide. Peach originated in Southwest China over 2,000,000 years ago and has since undergone thousands of years of domestication and improvement [1]. In the final centuries B.C., Chinese peach germplasm was first dispersed westwards to Persia and Europe via the ancient Silk Road and then was taken to the Americas  [1–4]). Almost all modern peach cultivars contain the genetic background of Chinese peach germplasm [2]. Extant peach germplasm exhibits diverse phenotypic traits due to its long evolutionary history, and the trait diversity results from variations in the genome. Due to its small genome size, economic and nutritional importance, and short reproductive cycle [5], peach has become an important model tree species for plant genetics and development.

The reference genome of the western rootstock peach ‘Lovell’ has been generated  [6] and was improved in 2017 [7]. With the rapid development of third-generation sequencing technology, more than one *de novo*-assembled genome has been released. The ‘Longhuashuimi’ (LHSM) peach genome has been assembled, and genetic loci regulating fruit flavour have been analysed [8]. The genome of ‘Rui You Pan1’ (RYP1) has also been generated, from which a 1.67-Mb causal inversion of flat-fruit shape was revealed [9]. The genome of ‘Chinese Cling’, an important founder cultivar, has also been released, and the fruit volatile contents associated with different domestication loci have been investigated [10]. Additionally, the genome of ‘Zhongyoutao 14’ (CN14), a temperature-sensitive semidwarf cultivar, has been assembled, and key genes controlling flower type and temperature-sensitive semidwarfism were identified [11].

Landraces are broadly defined as plant and animal species that are not improved by formal breeding and that are mainly maintained in their areas of origin. The natural biodiversity of landraces may serve as a raw resource for improving food crop flavour, quality, resistance to disease and adaptability to stressful environments. For example, an analysis of five Sudanese sorghum landraces revealed high contents of zinc (Zn), iron (Fe), phenolics and gluten, indicating that they could be used in the improvement of new value-added crops [12]. Chinese wheat landraces have higher grain Zn and Fe concentrations than wheat cultivars have and can thus serve as a potential genetic resource for enhancing grain mineral levels in modern wheat cultivars [13]. Notably, a locus regulating prolificacy was revealed in the prolific maize landrace ‘Sikkim Primitive’ [14]. Research on landraces is important for understanding plant genetics, trait regulation mechanisms and the breeding process.

Fei Cheng peach has a cultivation history of more than 1200 years and mainly includes two cultivars, ‘Fei Cheng Hong Li’ (FCHL) and ‘Fei Cheng Bai Li’ (FCBL), which were collected as representative local varieties and categorized as belonging to a landrace group during the phylogenetic analysis of 84 peach accessions in a previous study [15]. Due to the influence of the potential *S* gene, Feicheng peach has strict self-pollination characteristics [16], and its genome cannot be integrated with the genomes of other peach cultivars, leading to a homozygous genome for this peach. As an ancient landrace, Fei Cheng peach is expected to have distinct genomic differences from modern cultivated varieties. Here, we present a high-quality genome of the representative landrace FCHL obtained using high-depth PacBio long-read data complemented with Illumina short-read data and Hi-C sequencing data. Combining these approaches with RNA-seq technology, we identified candidate genes that regulate late florescence and narrow leaves. The FCHL genomic sequences will enrich the diverse genome collection of peach germplasm resources and advance the understanding of peach genetics and development.

## Results

### Genome sequencing and assembly

**Fig. 1:**
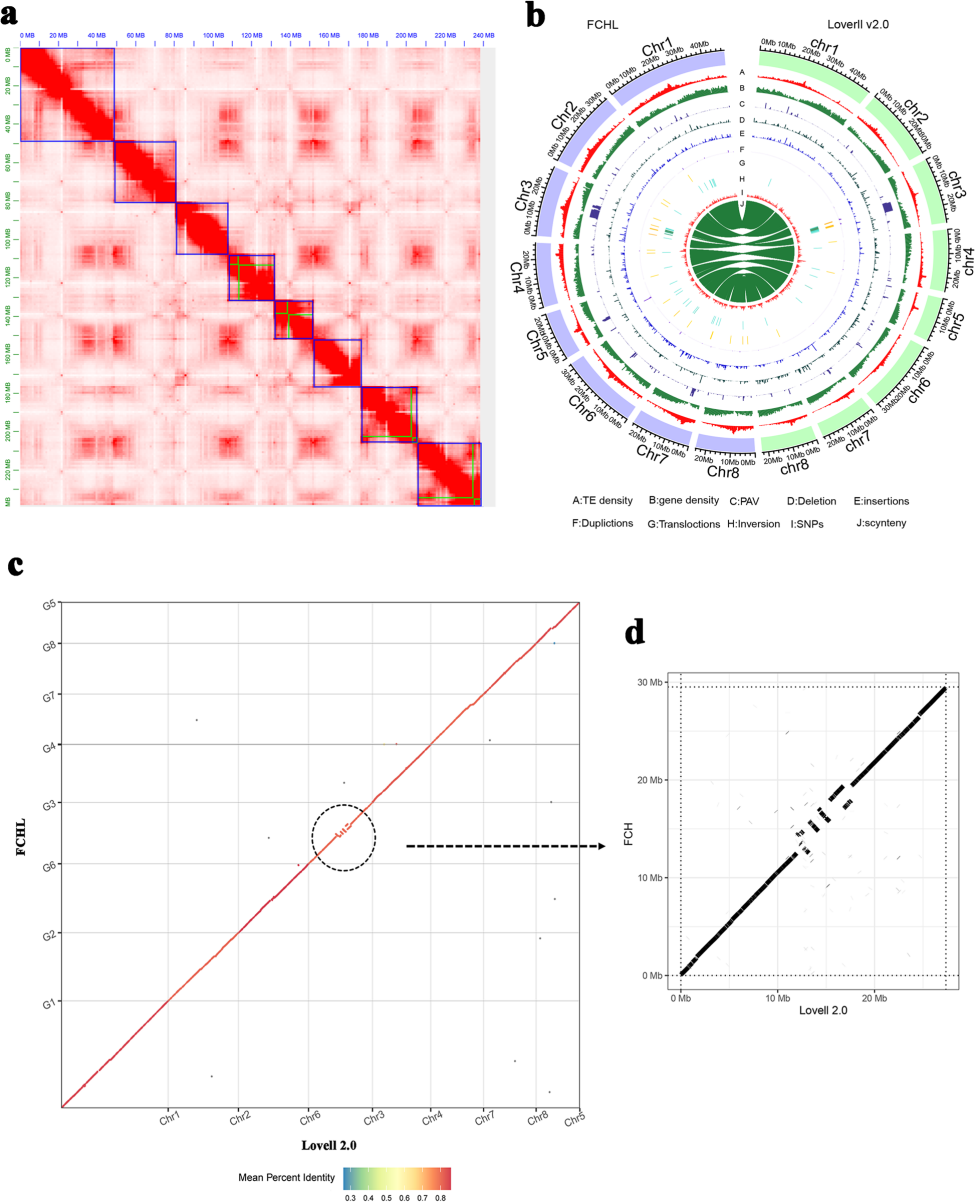
Characterization of the FCHL genome. **a** Hi-C interactions among eight chromosomes. Strong interactions are indicated in dark red, and weak interactions are indicated in white. The blue box represents the scaffold, and the green box represents the assembled contig. **b** Genomic variations between the FCHL (left) and ‘Lovell’ v2.0 (right) genome assemblies; A-J, TE density, gene density, PAVs, deletions, insertions, duplications, translocations, inversion, SNPs, and synteny. **c** Collinearity analysis of the FCHL and ‘Lovell’ v2.0 genomes. **d** The area of noncollinearity on Chr3: 10–20 Mb

The genome of FCHL was *de novo* assembled using 60× coverage of 18.22 Gb of HiFi reads, 62× coverage of 18.46 Gb of Illumina short reads and 93× coverage of 27.97 Gb of Hi-C data. Based on a *k*-mer analysis (*k* = 27) using all Illumina reads. HiFi reads were used to assemble the FCHL genome with the software hifiasm v0.14.2, generating a total of 789 contigs. The maximum contig length was 49.32 Mb, and the contig N50 was 26.93 Mb, which was 90-fold longer than that previously reported for the reference genome of ‘Lovell’ (N50, 294 kb). Fastp v 0.20.0 software was used to filter out the low-quality raw data from the 29.57 Gb of Hi-C data. A total of 27.97 Gb of clean data (94.67%) was maintained to anchor the contigs to eight pseudochromosomes with Juicer v1.5.7 software. In particular, only 8 scaffolds composed of 12 contigs were achieved in the assembly of the whole FCHL genome. In detail, 4 scaffolds composed of 4 individual contigs completely covered the lengths of chromosomes 1, 2, 4 and 7, and another 4 scaffolds composed of 8 contigs finished the assembly of the remaining 4 chromosomes (3, 5, 6 and 8) with 4 gaps (Fig. 1a; Table 1). The maximum scaffold size was 49.32 Mb, and the scaffold N50 was 29.51 Mb. Finally, a chromosome-level genome of 239.06 Mb was generated (Fig. 1b). Compared to the ‘Lovell’ v2.0 genome, this new genome showed very few gaps (4 gaps) and an extralong contig N50 (26.93 Mb) (Table 1), showing contiguous splicing and accurate assembly during sequencing.

**Table 1:** Parameter statistics of the FCHL genome assembly and the Lovell v2.0 reference genome

**Genomic feature**	**FCHL**^**a**^	**Lovell v2.0**^**b**^
Total assembly size (Mb)	239.06	227.4
Number of contigs	789	2525
Largest contigs (Mb)	49.32	1.5
Contig N50	26.93Mb	255.4 kb
Number of scaffolds	12	191
Number of gaps	4	1828
Largest scaffolds (Mb)	49.32	47.85
Scaffold N50 (Mb)	29.51	27.37
Sequences anchored to chromosomes (Mb)	234.3	225.7
Repetitive sequences (%)	35.55	44.85
LTR assembly index, LAI	28.21	21.29
Protein-coding genes	28,645	31,972
Transcripts	58,360	47,089
Complete BUSCO (%)	98.1	97.6
GC content (%)	38.95	37.0

^a^Parameter statistics of the FCHL genome were analyzed in this study

^b^Parameter statistics of the Lovell v2.0 genome were from the previous study [7]

### Assessment of genome quality

The quality of the assembled FCHL genome was assessed using three strategies. First, the generated Illumina short reads covered 98.02% of the genome, showing the structural completeness and accuracy of the assembled genome. Second, the long terminal repeat (LTR) assembly index (LAI) [17] was used to evaluate the quality of the FCHL genome assembly, which exhibited a high LAI score (28.21) reaching the “gold standard level” (Fig. S3) and exceeding that of the ‘Lovell’ v2.0 genome (21.29). Third, approximately 98.1% of the BUSCO genes could be aligned to the genome assembly, showing a higher completeness level than the ‘Lovell’ v2.0 genome (96.8%). Overall, these lines of evidence supported the high quality of the assembled FCHL genome.

To further assess the accuracy of the FCHL genome assembly, collinearity analysis between the assembled FCHL genome and the ‘Lovell’ reference genome was also carried out with Minimap2 software (Fig. 1c; Table 2) [16, 18]. The results showed good collinearity of the two genomes along the eight chromosomes and 1563 syntenic regions, which contained 194.81 Mb (81.49%) of FCHL genome sequences and 197.03 Mb (86.64%) of ‘Lovell’ genome sequences. However, there was a distinct area of noncollinearity in the middle of chromosome 3 (Fig. 1d), which experienced a recombination event [9] and was previously detected in chromosome alignments of LHSM vs. ‘Lovell’ and ‘Chinese Cling’ vs. ‘Lovell’.

**Table 2:** Summary of genomic variations between the FCHL and Lovell 2.0 genomes

Structural annotations			
Variation_type	Count	Length_ FCHL	Length_ Lovell
Syntenic regions (bp)	1563	194807210	197037302
Inversions (bp)	28	3357350	3121614
Translocations (bp)	493	5001688	4845262
Duplications (FCHL)	2140	12993722	-
Duplications (Lovell)	142	-	1965632
Not aligned (FCHL)	3021	24259394	-
Not aligned (Lovell)	1475	-	21816058
Sequence annotations			
Variation_type	Count	Length_ FCHL	Length_ Lovell
SNPs	432535	432535	432535
Insertions	62393	1830249	-
Deletions	38852	-	2044082
Copygains	117	703306	-
Copylosses	115	-	691708
Highly diverged	2565	8618641	10355233
Tandem repeats	37	429945	273300

### Genome annotation

The EDTA pipeline was used for repeat sequence annotation. We detected a total of 84.99 Mb of repeat sequences (35.55%) in the FCHL genome (Table 2), which included 81.01 Mb of transposable elements (TEs) (33.51%) and 3.98 Mb of simple sequence repeats (SSRs) (2.04%). Compared with the ‘Lovell’ genome (29.60%) [7], the FCHL genome harboured more TEs. The TEs mainly consisted of three types, LTRs (17.7%), terminal inverted repeats (TIRs) (13.06%) and Helitrons (2.74%). Copia and Gypsy LTRs were the two main LTR-type transposon types, and their insertion times in the genome were analysed (Figs. S4-6; Table S2). The results showed that almost all Copia and Gypsy TEs were formed within 1 Mya, representing an active period of FCHL genome reset.

We carried out gene prediction using an integrative strategy combining homologous prediction and *ab initio* prediction. Homologous prediction was carried out based on 7 protein libraries for related species and RNA sequencing of 6 FCHL tissues (details in the Methods section), and *ab initio* prediction was performed by integrating the results of homologous prediction with MARKER software to generate the final gene set. A total of 28645 genes and 58360 proteins were annotated. Of the annotated transcripts, the BUSCO completeness was 93.6%, suggesting comprehensive and complete annotation. Then, 9 public databases were used for functional annotation of proteins (Figs. S7-12; Table S3), and 95.59% (55784/58360) of these transcripts could be annotated by at least one database.

In addition, we annotated the noncoding RNA (ncRNAs) in the FCHL genome. A total of 131 miRNAs were detected in the FCHL genome—fewer than were detected in the ‘Lovell’ genome (189 miRNAs). We also detected 369 small nucleolar RNA (snoRNAs), 114 small nuclear RNAs (snRNAs), 1383 ribosomal RNAs (rRNAs) and 495 tRNAs in the FCHL genome.

### Variations between the FCHL and ‘Lovell’ 2.0 genomes

**Fig. 2:**
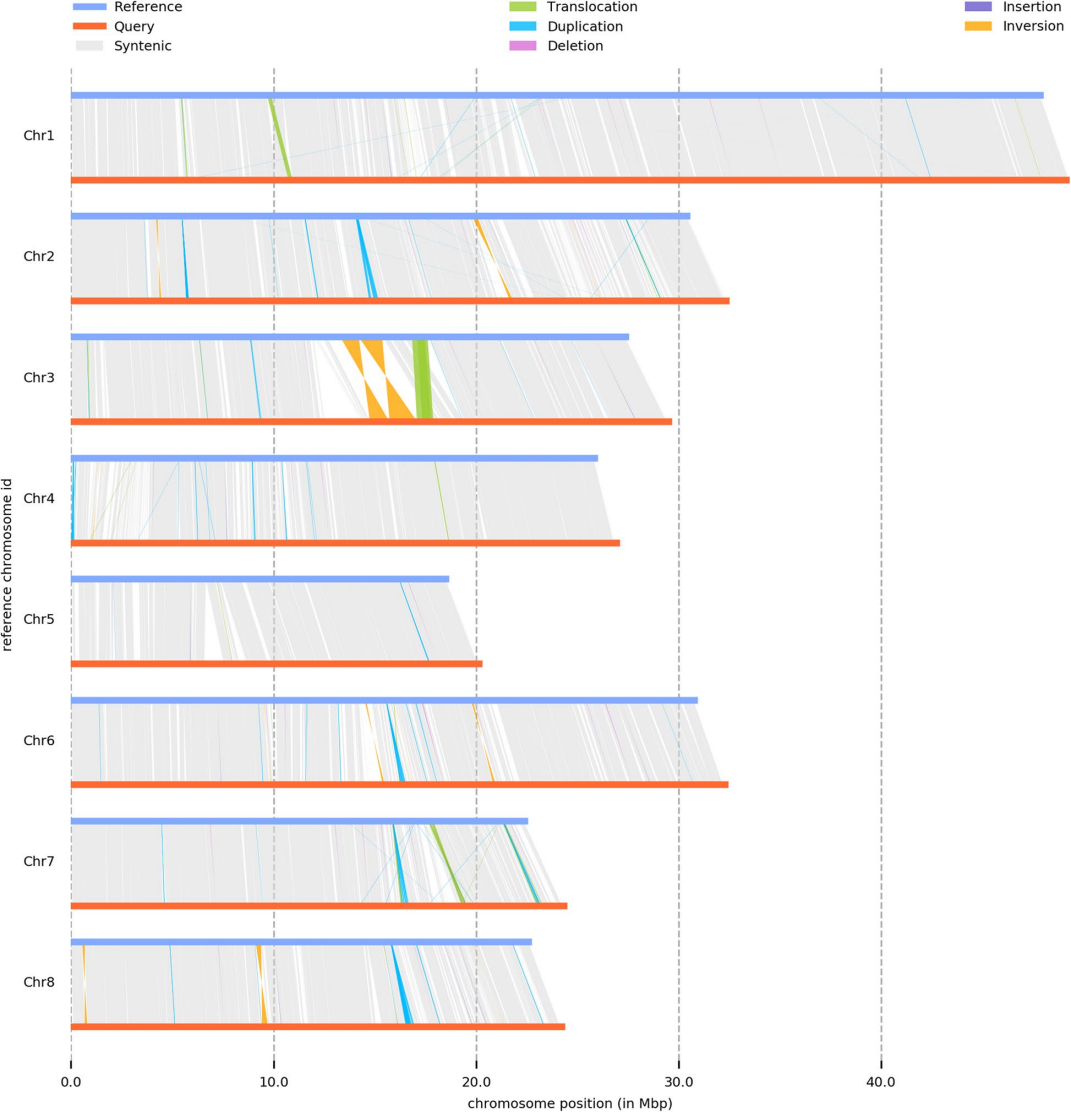
The structural variations between the FCHL (Query) and ‘Lovell’ v2.0 (reference) genomes. Different types of structural variation (translocation, duplication, inversion, deletion and insertion) are shown in different colours

Some structural variants (SVs) in peach have been found to contribute to distinct phenotypic variations, such as fruit shape [9] and fruit pubescence [19]. Thus, analysing the SVs between different genomes is especially important for identifying the functional genes regulating phenotypes and for providing an overview of the origin of peach varieties. FCHL, an ancient landrace from China, produces juicy peaches and shows strict self-compatibility during flower pollination, and its phenotypes are more similar to those of cultivated varieties than to those of wild peaches, whereas ‘Lovell’ produces acidic fruit, has strong resistance and is now widely used as a rootstock in Europe and America [20]. Although the phenotypic differences between the two varieties are obvious, to further reveal hidden genomic variations and explore the unique genomic areas of FCHL as a landrace, we aligned the FCHL genome to the ‘Lovell’ reference genome with the software Sryi [21] and identified 432535 SNPs, 62392 insertions, 38852 deletions and 7299 SVs (Fig. 2; Table 2). We also detected 1475 Lovell-specific genomic segments (21.81 Mb) and 2288 FCHL-specific genomic segments (24.26 Mb). The results clearly showed different types of SVs, including inversions, translocations, duplications, deletions and insertions, between the two genomes. Large-fragment inversions were mainly distributed on chromosomes 2, 3 and 8 (Fig. 2). The two largest inversions (0.96 Mb and 1.35 Mb) were distributed adjacently in the 14.76 Mb - 17.09 Mb region of chromosome 3, which was primarily responsible for the area of noncollinearity detected in collinearity analysis. The detected large-fragment translocations were mainly distributed on chromosomes 1, 3 and 7 (Fig. 2). The largest translocation (0.69 Mb) was also distributed in the area of noncollinearity on chromosome 3. The largest duplication was detected at 16.60 Mb-16.75 Mb on chromosome 8. The largest deletion (17.5 kb) was detected in the terminal part of chromosome 5, and the largest insertion (18.8 kb) was detected at 19.51 Mb-19.52 Mb on chromosome 7. The two genomes exhibited a large number of genomic variations upon alignment, which could affect gene structure.

**Fig. 3:**
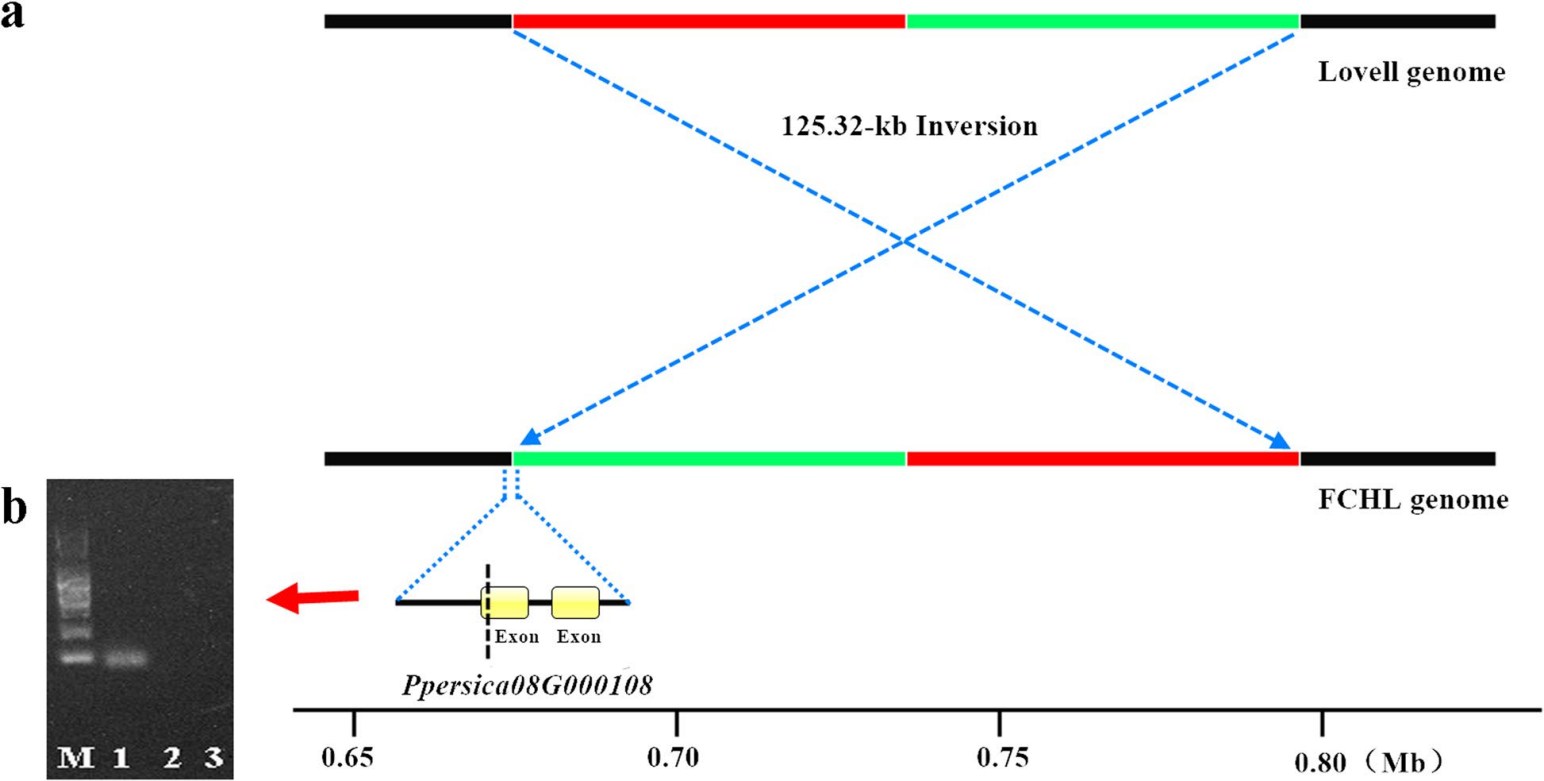
Schematic diagram of a 125.32-kb inverted fragment (**a**) in the FCHL genome compared with the reference genome Lovell and PCR confirmation of the variation in the FCHL gene *Ppersica08G000108* (**b**). M, marker; 1, FCHL; 2, Lovell; 3, the negative control

For example, a 125.32-kb inversion was detected at the initiating terminus (0.68 Mb-0.80 Mb) of chromosome 8 (Fig. 3a), and one terminus of the inversion emerged in an exon of the FCHL gene *Ppersica08G000108* (confirmed by PCR) (Fig. 3b), which was annotated as a universal stress protein A-like protein (USP). The terminus of the 125.32-kb inversion occurring in the exon could destroy the integrity of the putative resistance gene *Ppersica08G000108*.

### Gene family analysis of ten species

**Fig. 4:**
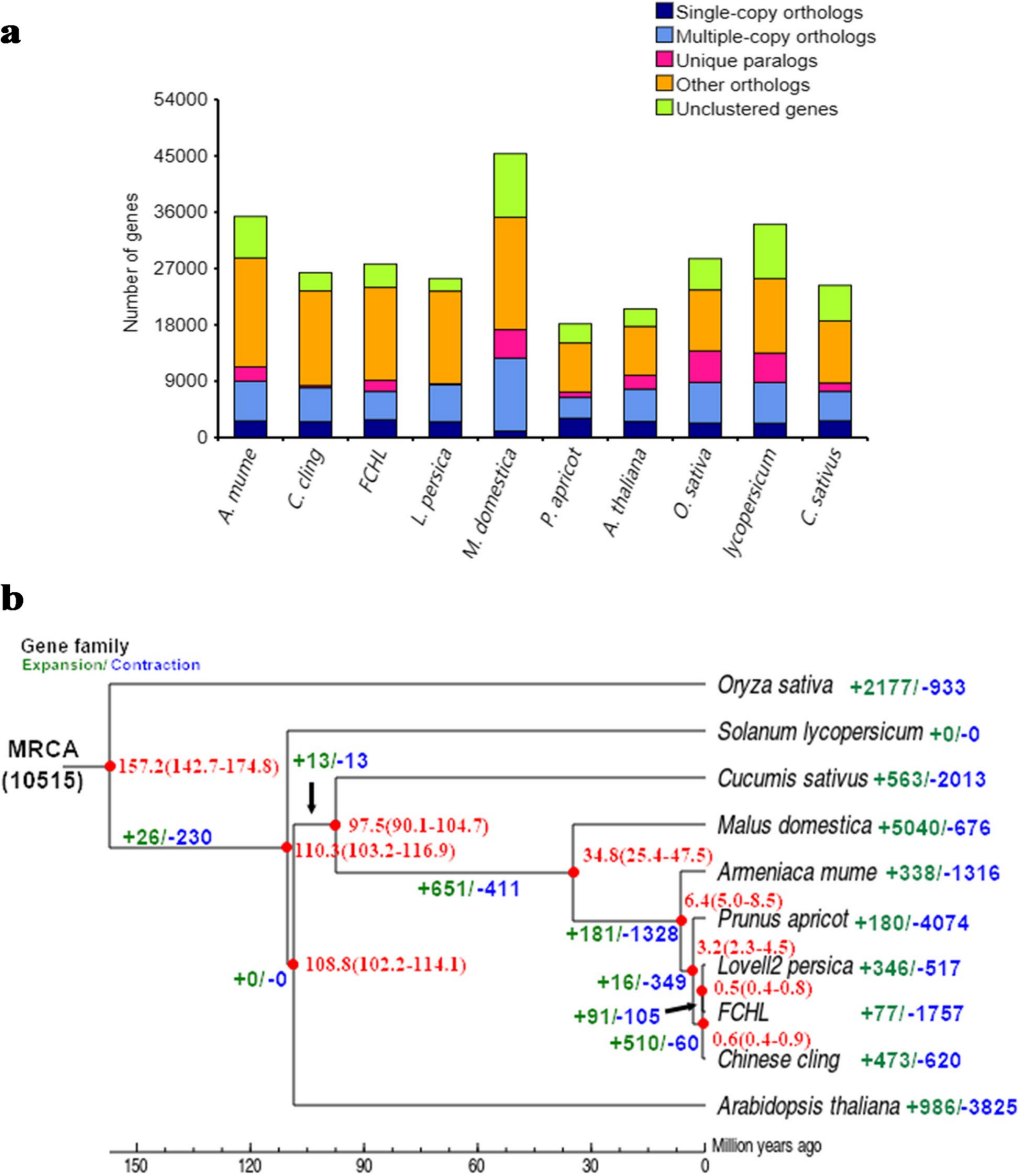
Gene family analysis of FCHL and nine other species. **a** The statistical results for homologous gene numbers of ten species. **b** Estimation of the divergence time and gene family expansion and contraction of 10 species. MRCA, most recent common ancestor. Red numbers indicate the divergence time. Green numbers indicate the number of expanded gene families, and blue numbers indicate the number of contracted gene families during the evolution of species

To analyse the evolution of FCHL at the genome level, we added another 9 genomes, including the peach reference genome variety ‘Lovell’ (v2.0), the juicy peach founder ‘Chinese Cling’, the drupaceous fruit species *Prunus apricot* and *Armeniaca mume* Sieb., the nondrupaceous fruit *Malus domestica*, and the non-Rosaceae plants *Arabidopsis thaliana*, *Solanum lycopersicum*, *Cucumis sativus*, and *Oryza sativa* (serving as the outgroup) (Fig. 4a). A phylogenetic tree with divergence times of the 10 species was constructed based on 4071 single-copy gene families. Three peach varieties and two close relatives, *Prunus apricot* and *Armeniaca mume* Sieb, were expectedly clustered into a single group (Fig. 4b) that had a distant evolutionary relationship with the other five genomes. Compared with the other two peach genomes, the FCHL genome contained more unique paralogues (Fig. 4a). The divergence time of ‘Chinese Cling’ (approximately 0.6 Mya) was earlier than that of FCHL and ‘Lovell’ (approximately 0.5 Mya), indicating that ‘Chinese Cling’ was domesticated earlier than FCHL and ‘Lovell’ and confirming that ‘Chinese Cling’ was a founder cultivar in peach breeding.

There are distinct differences in the expanded and contracted gene families among the ten genomes. The genome with the largest number of expanded gene families was *Malus domestica*, and the genome with the largest number of contracted gene families was *Prunus apricot*. Among the three peach genomes, the FCHL genome had the smallest number of expanded families (77) and the largest number of contracted gene families (1757) (Fig. 4b). We selected 61 gene families with significant expansion (321 genes) and 1282 gene families with significant contraction (1637 genes) (*p*<0.05) for Kyoto Encyclopedia of Genes and Genomes (KEGG) enrichment analysis (Figs. S13-14). The pathways of homologous recombination and sesquiterpenoid and triterpenoid biosynthesis were enriched in the expanded genes, while the pathways of phenylalanine metabolism, isoquinoline alkaloid biosynthesis and ABC transporters were enriched in the contracted genes. Alkaloids and related synthetic material were mainly enriched in the contracted gene families.

### Genes related to flower development in the FCHL genome

**Fig. 5:**
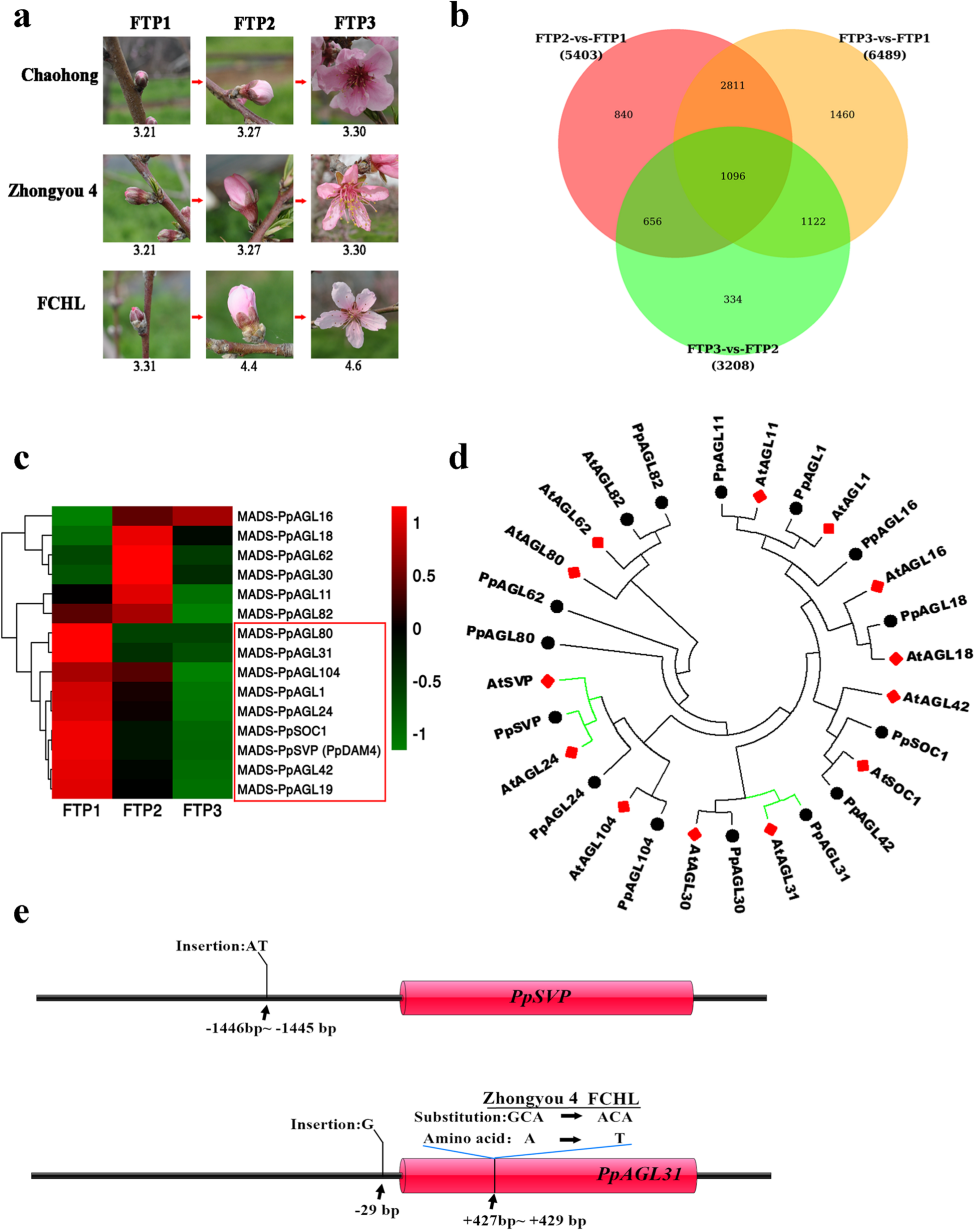
Late florescence of FCHL and related regulatory genes. **a** Florescence comparison of FCHL and two other varieties, ‘Chaohong’ and ‘Zhongyou 4’, during three phases. FTP1, red dot period; FTP2, budding flower period; FTP3, full bloom period. The numbers represent the dates of the three phases of flowering. **b** Common and unique differentially expressed genes among different comparison groups. **c** Fifteen differentially expressed *MADS* genes at three flowering stages (FTP1, FTP2 and FTP3). The 9 *MADS* genes marked by the red box were mainly expressed in FTP1. **d** Phylogenetic tree of 15 differentially expressed *MADS* genes in FCHL and *Arabidopsis*. e The variations of *PpSVP* and *PpAGL31* in FCHL vs. ‘Zhongyou 4 ’

According to several years of field observations, the FCHL florescence time was distinctly later than that of most other peach varieties in the Taian area. For example, the flower bud red dot and full bloom periods of the peach varieties ‘Zhongyou 4’ and ‘Chaohong’ (originating from America) were March 25^th^ and March 30^th^ in 2022, respectively, whereas those of FCHL were April 1^st^ and April 6^th^ (Fig. 5a), showing a florescence delay of one week in this landrace.

To explore the late florescence in FCHL, we performed RNA-seq analysis based on the assembled FCHL genome. RNA was extracted from FCHL flowers at three phases: FTP1 (the red dot period), FTP2 (the budding flower period) and FTP3 (the full bloom period). A total of 60.90 Gb of clean data was generated, and an average of 96.12% reads were mapped to the assembled FCHL genome (Table S4). A total of 8319 differentially expressed genes (|log_2_FoldChange| ≥1, *P* values < 0.05) were identified across the three phases of flower development (Fig. 5b). In this analysis, 15 *MADS-box* genes were differentially expressed during flower development (Fig. 5c). Nine *MADS-box* genes, *PpAGL80*, *PpAGL31*, *PpAGL104*, *PpAGL1*, *PpAGL24*, *PpSOC1*, *PpSVP* (*PpDAM4*), *PpAGL42* and *PpAGL19,* were highly expressed in FTP1 and showed rapidly decreasing expression in FTP2 and FTP3. A known inhibitor, *PpSVP* (*PpDAM4*), was detected in FTP1, indicating that the late florescence of this landrace was closely related to *PpSVP* (*PpDAM4*). The similarity of protein structure generally indicates the consistency of function. A phylogenetic tree of 15 *MADS-box* FCHL proteins and their orthologs in *Arabidopsis* was analysed, and PpAGL31 showed a close relationship with AtAGL31, a repressor of flowering in *Arabidopsis* (Fig. 5d). Compared with the *PpAGL31* and *PpSVP* of ‘Zhongyou 4’, some base insertions were detected in the FCHL promoters and an amino-acid substitution was found in the FCHL PpAGL31 (Fig. 5e). These data combined with the expression data indicated that *PpSVP* and *PpAGL31* mainly played a role in FTP1 and could influence florescence, which were good candidate genes for the regulation of florescence in FCHL.

To gain an overview of functional changes in the transcriptome during flower development, we performed KEGG enrichment analysis (Figs. S15-16). The results showed that the cell cycle, plant hormone signal transduction, carotenoid biosynthesis, phenylpropanoid biosynthesis, and starch and sucrose metabolism pathways were distinctly enriched from FTP1 to FTP2, and the plant hormone signal transduction, galactose metabolism, flavonoid biosynthesis, phenylpropanoid biosynthesis, and starch and sucrose metabolism pathways were distinctly enriched from FTP2 to FTP3, which mainly supported the flower development process.

### Narrow leaf-related genes in the FCHL genome

**Fig. 6:**
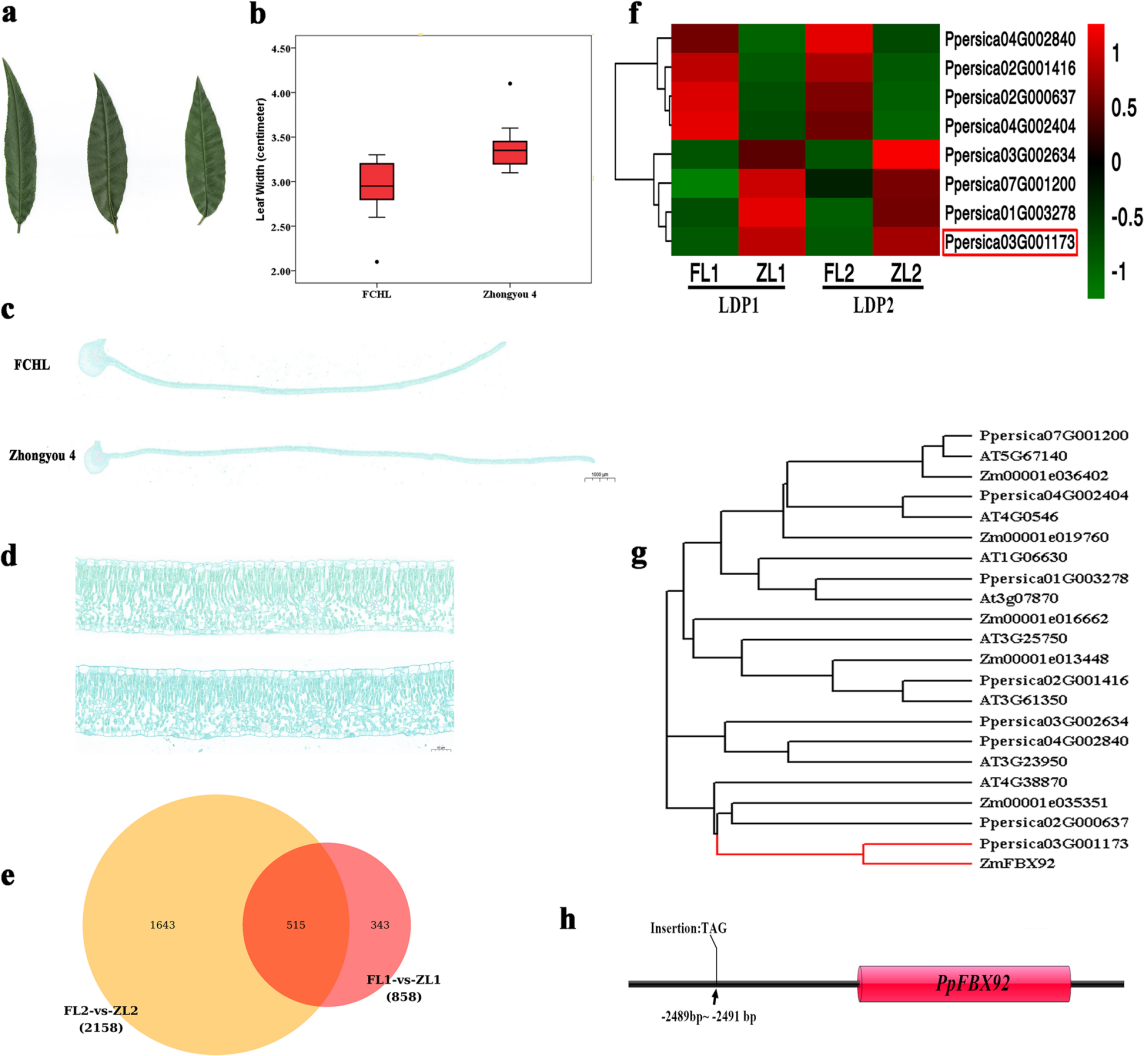
The narrow leaves of FCHL. **a** The leaf phenotype of FCHL and two other varieties, ‘Chaohong’ and ‘Zhongyou 4’. **b** Box plot of leaf width data from FCHL and ‘Zhongyou 4’. The middle line of the box represents the median of the data; the upper and lower limits of the box represent 25% and 75% of the whole population, respectively; and black dots represent outliers. **c** Paraffin section of the half section of the transverse section of the leaf centreline under a microscope (1*); Scale, 1000 μm; upper, FCHL leaf; lower, ‘Zhongyou 4’ leaf. **d** Paraffin sectioning of the half section of the transverse section of the leaf centreline under a microscope (40*); Scale, 50 μm; upper, FCHL leaf; lower, ‘Zhongyou 4’ leaf. **e** Common and unique differentially expressed genes between two developmental periods, the young leaf period and the mature leaf period. FL1 and ZL1, samples of FCHL and ‘Zhongyou 4’ leaves at the young leaf period, respectively; FL2 and ZL2, samples of FCHL and ‘Zhongyou 4’ leaves at the mature leaf period; **f** 8 differentially expressed *F-box genes* at the two development periods, LDP1 (young leaf period) and LDP2 (mature leaf period); FL1 and ZL1, samples of FCHL and ‘Zhongyou 4’ leaves at the young leaf period, respectively; FL2 and ZL2, samples of FCHL and ‘Zhongyou 4’ leaves at the mature leaf period. **g** Phylogenetic tree of 8 differentially expressed *F-box* genes among FCHL, *Arabidopsis* and maize. h The variations of *PpFBX92* in FCHL vs. ‘Zhongyou 4 ’

Another obvious unique trait of FCHL is its narrow leaves. Compared with the leaves of the peach varieties ‘Zhongyou 4’ and ‘Chaohong’, those of FCHL are much narrower (Fig. 6a-b). We also analysed this difference at the cellular level with a paraffin sectioning experiment (Fig. 6c). The results showed no significant difference in the cell expansion of mature leaf longitudinal sections between FCHL and ‘Zhongyou 4’ (Fig. 6d; Table S6). Therefore, the narrowed leaf phenotype of FCHL compared with ‘Zhongyou 4’ was caused by a sharp decrease in the number of mesophyll cells.

To further explore the regulators of narrow leaves, comparative transcriptome analysis of FCHL and ‘Zhongyou 4’ leaves during two developmental periods, LDP1 (the young leaf period) and LDP2 (the mature leaf period), was carried out based on the assembled FCHL genome. A total of 81.98 Gb of clean data was obtained from 12 sequenced samples (Table S5). The effective dataset size of each sample was distributed from 6.37 Gb to 7.28 Gb, and the rates of read alignment to the reference genome ranged from 95.38% to 97.52%. A total of 858 and 2158 differentially expressed genes (|log_2_FoldChange| ≥1, *P* values < 0.05) were identified between FCHL and ‘Zhongyou 4’ leaves at the young and mature periods, respectively, and 515 differentially expressed genes were detected in both periods (Fig. 6e). Reported* F-box genes,* such as *AtFBL17*, *ZmFBX92*, and *AtFBX92*, play an important role in regulating the development of leaf size [22, 23]. This category of genes showed a close relationship with leaf development. Among the 515 differentially expressed genes, we found that 4 *F-box* genes were highly expressed in young and mature FCHL leaves, and another 4 *F-box* genes were highly expressed in young and mature ‘Zhongyou 4’ leaves (Fig. 6f). A phylogenetic tree of 8 *F-box* FCHL proteins and their orthologs in *Arabidopsis* and maize was analysed, and PpFBX92 (Ppersica03G001173) showed a close relationship with ZmFBX92 (Fig. 6g), a positive regulator of leaf size. Compared with the *PpFBX92* of ‘Zhongyou 4’, three base insertions were detected in the FCHL promoter (Fig. 6h).As an orthologous gene of *ZmFBX92*, *PpFBX92* could serve as a good candidate gene for studies on leaf development.

## Discussion

*De novo* genome assembly of representative and characteristic peach varieties is an important strategy that has contributed to understanding various phenotypes and revealing functional genes [8–11]. Cultivated plants undergo morphological evolution in multiple steps. For example, wild tomato species (*Solanum pimpinellifolium*), originating in South America, evolved into cherry tomato groups (*Solanum lycopersicum* var*. cerasiforme*) after exposure to environmental stress and animal selection and finally gave rise to cultivated tomato accessions in response to artificial improvement and targeted breeding [24]. Peach also has a two-step evolutionary history involving early natural selection and late artificial improvement [1, 15]. Available evidence reveals that peach endocarp fossils appear in the record at 2.6 Mya [25], and the earliest history of domestication dates back to 7500 years ago in the Yangzi River valley of southern China [26]. Landrace peaches could be seen as an intermediate group between wild species and derived modern cultivars. In a phylogenetic analysis of 84 peach accessions [15], the branch bearing FCHL was located closer to that of the wild group than to that of the cultivated group. It was conjectured that FCHL mainly underwent a long period of early natural selection rather than improvement by artificial breeding because of its strict self-pollination. Our study suggests that the insertion times of LTR transposons into the FCHL genome were concentrated within 1 Mya, indicating that the active period of FCHL genomic variation coincided with the natural selection period. The FCHL genome did not suffer a narrowed genetic background caused by artificial breeding, which could also be explained by some of its conserved distinctive phenotypes, e.g., late flowering, narrow leaves and strict self-pollination. In this study, to obtain a comprehensive understanding of the landrace FCHL, we *de novo* assembled its genome and generated a high-quality genome. As revealed by genome assembly quality assessment, compared with the reference genome ‘Lovell’ v2.0, the FCHL genome has a higher ratio of short sequences anchored to chromosome size, a higher LAI score, a longer contig N50 and a higher BUSCO score. In particular, we utilized only 8 scaffolds (12 contigs) to complete the genome assembly; in other words, each utilized scaffold was a whole chromosome. A long contig N50 indicated that the splicing and assembly process was easy and accurate. Moreover, there were only 4 gaps in the FCHL genome assembly, which was far fewer than the number in the ‘Lovell’ genome (1828 gaps). Very few generated gaps indicated that the FCHL genome did not contain many equivocal chromosome areas, and we inferred that this finding might be related to its strict self-pollination, which reduced the probability of introgression from exogenous genomes and chromosomal recombination events. Overall, the assembled FCHL genome showed good continuity and completeness and could serve as a reference genome for understanding genetic mechanisms, evolutionary processes and molecular breeding.

The analysis of the FCHL gene families showed that the pathways in which the expanded gene families were enriched were those of homologous recombination and sesquiterpenoid and triterpenoid biosynthesis. Sesquiterpenoids and triterpenoid biosynthesis are widely distributed in plants, and most of them have strong aromas and biological activity [27]. The expanded gene family in which the sesquiterpenoids and triterpenoid biosynthesis pathways were enriched provided insight into the rich aroma of FCHL fruit. Moreover, alkaloids and related synthetic material were enriched in the contracted gene families. Alkaloids can increase the antibacterial function of plants and make them resistant to invasion by microorganisms [28]. The observed gene family contraction provided clues for the declining adaptation to the external biological environment of FCHL. The functions of some FCHL resistance genes were inhibited or inactivated, but the functions of genes related to fruit aroma and sweetness were enhanced compared with those in the rootstock ‘Lovell’, and these candidate genes could advance our understanding of individual diversity at the genomic level.

*MADS-box* genes are widely found to be related to various aspects of flower development and formation [29], and peach dormancy is controlled by *MADS-box* genes [30]. Combining the RNA-seq databases and phylogenetic tree, we finally identified two key negative *MADS-box* regulators, *PpSVP* (*PpDAM4*) and *PpAGL31*, showing a close relationship with the dormancy of flower buds. Previous studies have shown that *dormancy-associated MADS-box* (*DAM*) genes serve as key regulators of peach dormancy and that *DAM* 1-6 show different functions in the regulatory process [30–33]. *PpSVP* (*PpDAM4*) plays an important role in controlling dormancy and chilling requirements in peach floral buds [33]. Although *PpAGL31* is a novel gene and its function in the regulation of flowering development has not been reported in peach, we could infer its function on the basis of its orthologous gene *AtAGL31* (AtMAF2), a repressor of flowering at low temperature via interaction with *AtSVP* [34], and *PpSVP* (*PpDAM4*) and *PpAGL31* were likely located in the same regulatory pathway. Compared with those in other peach varieties, the negative regulators PpSVP (PpDAM4) and PpAGL31 showed slower degradation in FCHL at the same temperature, and this dosage effect of the two negative regulators was the dominant reason for the late florescence of FCHL. We inferred that some degradation enzymes, such as ubiquitinases, exist in FCHL that yield a higher chilling requirement to decrease the negative regulators PpSVP (PpDAM4) and PpAGL31. For the other 7 *MADS-box* genes highly expressed in FTP1, all of their orthologous genes in *Arabidopsis thaliana* showed positive roles in flower development, which means that they might not determine florescence and bud dormancy. In summary, the genes regulating peach florescence were further enriched and could provide a foundation for research on the molecular mechanisms of peach dormancy and flower development.

## Conclusions

In this study, an assembled high-quality genome of the landrace FCHL was obtained that displayed genomic details. Some candidate genes related to resistance, florescence and leaf size were identified, providing a basis for further research on these traits.

## Methods

### Plant materials and sampling

The landrace peach FCHL was collected from Feicheng city, Shandong Province, China (116.77°E, 36.18°N) and was maintained in the field under normal cultural conditions at the Tianping Lake experimental base, Shandong Institute of Pomology, Taian city, Shandong Province, China (117.08°E, 36.20°N). FCHL was propagated by bud grafting onto Ye Mao Tao rootstock in 2009.

To identify genes regulating flower development, FCHL flower samples at three development periods, FTP1 (flower time period 1: red dot period), FTP2 (flower time period 2: budding flower period) and FTP3 (flower time period 3: full bloom period), were collected. Three biological replicates were collected for each sample.

To identify genes regulating leaf shape, FCHL and ‘Zhongyou 4’ leaf samples at two developmental periods, LDP1 (young leaf period) and LDP2 (mature leaf period), were collected. Three biological replicates were collected for each sample.

### RNA-seq

RNA purity was checked using a NanoPhotometer spectrophotometer (IMPLEN, CA, USA). The libraries were constructed using the TruSeq Stranded mRNA LT Sample Prep Kit (Illumina, San Diego, CA, USA) according to the manufacturer’s instructions and sequenced on the Illumina sequencing platform (HiSeqTM 2500 or Illumina HiSeq X Ten).

For genome annotation, total RNA was extracted from 6 tissues (roots, stems, leaves, flowers, young fruits and mature fruits) of FCHL. For PacBio isoform sequencing (Iso-Seq), libraries were constructed with the BluePippin Size Selection System protocol and the Clontech SMARTer PCR cDNA Synthesis Kit. RNA from 6 tissues (roots, stems, leaves, flowers, young fruits and mature fruits) of FCHL was mixed and then sequenced on a PacBio Sequel instrument at Shanghai OE Biotech Co., Ltd. (Shanghai, China).

### Genome library construction and sequencing

For the genome survey, genomic DNA was extracted from leaves of FCHL *via* the cetyl trimethylammonium bromide method (Murray and Thompson, [35]) and sheared into fragments with a length of -350 bp using S220 Focused ultrasonicators (Covaris, USA). The 3’ ends of the sheared fragments were ligated with adapters. The libraries were sequenced on the Illumina NovaSeq platform (Illumina Inc., USA) with 150-bp paired-end reads.

For PacBio sequencing, a library of -20 kb fragments was constructed based on the -20 kb SMRTbell Libraries Protocol. The genomic DNA of FCHL was assessed by standard agarose gel electrophoresis and Thermo Fisher Scientific Qubit fluorometry. The -15 kb DNA fragments were generated by using g-TUBE (Covaris) and then purified with 0.45× AMPure beads. The libraries were sequenced using P6-C4 chemistry on the PacBio Sequel II sequencing platform (PacBio) at Shanghai OE Biotech Co., Ltd. (Shanghai, China).

Damaged DNA and ends were enzymatically repaired as recommended by PacBio. Hairpin adapters were ligated using a blunt-end ligation reaction. The libraries were sequenced using P6-C4 chemistry on a PacBio Sequel II sequencing platform (PacBio) at Shanghai OE Biotech Co., Ltd. (Shanghai, China).

### Hi-C library construction and sequencing

For Hi-C library construction [36], the crosslinked nuclear DNA was digested, biotin-labelled and ligated to form chimeric circles, followed by pull-down and cut-off to construct the library. A library of -350 bp fragments was sequenced using the Illumina HiSeq X-Ten platform (Illumina, San Diego, CA, USA) for chromosome pseudomolecule construction.

### Genome size estimation

For the genome survey, the K-mer distribution was estimated with the program Jellyfish (parameters “-m 17 -C”) [37]. By using the program GenomeScope  [38], the size, heterozygosity rate and repeatability of the FCHL genome were estimated.

### Genome assembly and annotation

By using sequencing mode ccs (https://github.com/PacificBiosciences/ccs, version: 4.2.0), multiple subreads from the same SMRTbell molecule template were subjected to consistency correction, and a highly accurate consensus sequence (HiFi reads) was generated. A total of 18 Gb of high-quality HiFi reads were generated (read N50 23931 bp). For genome assembly, the FCHL genome was assembled with Hifiasm v 0.14.2 software (Cheng et al., 2020).

### Hi-C scaffolding and gap filling

By sequencing, a total of 198,065,842 raw reads were generated. Then, a total of 187,514,716 high-quality clean reads were retained, and fastp v 0.20.0 software [39] was used to filter out the repeat sequences, splice sequences and low-quality sequences from the raw reads. The clean reads were aligned to the contigs using BWA-mem v0.7.17 [40] with default parameters. The clean reads were input into the software Juicer v1.5.7 [41] and 3d-DNA v 20180922 [36] to apply the Hi-C analysis and scaffolding pipelines. *Via* Juicebox v 1.11.9 software [41], the hic contact matrix was visualized. Based on neighbouring interactions, the mistakes in the hic contact matrix during assembly and connection were manually corrected.

### Repeat annotation

To customize filtering scripts for *de novo* identification of each TE class (LTRs, TIRs and Helitrons), Extensive *de novo* TE Annotator11 (EDTA v 1.7.0) ([42] incorporating LTRharvest from genometools (version: 1.5.10) [43, 44], LTR_FINDER v 1.0.7 [45], TIR-Learner v 2.4 [46], and HelitronScanner v1.1 [47] was performed. Then, using the software LTR_retriever v 2.8.2 [17], RepeatModeller v 1.0.11 [48], and RepeatMasker v 4.0.9 [49], we eliminated the false positive results of LTRs and searched for undiscovered TEs in the genome to finish the comprehensive TE library. TE homologue annotation of the library was carried out with RepeatMasker and structural annotation from the raw step.

### Gene prediction and annotation

Reads from 6 tissues (roots, stems, leaves, flowers, young fruits and mature fruits) were aligned to the genome using HISAT2 v2.10.2 software [50], and then transcripts (RNA-seq) were reconstructed using StringTie v1.3.0 software. To promote the annotation of RNA-seq reads, a high-quality full-length transcript (Iso-seq) was generated based on equivalent mixtures of the extracted RNAs from the 6 tissues mentioned above. Proteomes of 7 species (*Fragaria vesca*, *Arabidopsis thaliana*, *Prunus persica*, *Malus* × *domestica*, *Prunus yedoensis*, *Prunus avium*, and *Prunus dulcis*) were downloaded from previously published articles or related databases.

To accurately annotate the protein-coding genes, two runs of MAKER v 2.31.10 [51] were carried out. In the first run of gene prediction, the assembled transcripts, homologous proteins and ESTs from the NCBI database were aligned to the reference genome with blast n/blast x and exonerate. Meanwhile, the software Augustus v 3.3.2 [52] and GeneMark-ES v 4.3.8 [53] were trained by BRAKER2 v 2.1.4 [54] during transcript alignment aligned to the genome. In the second run, we integrated the first round of homology prediction evidence with MAKER and carried out the second round of *de novo* gene prediction to obtain the final gene annotations using the software Augustus and GeneMark-ES. To evaluate annotation completeness, BUSCO v 3.1.0 was used [55].

According to the best match of the protein sequences in the NR, KOG, GO, Swiss-Prot, TrEMBL, eggnog, KEGG, InterPro and Pfam databases, gene functions were assigned with the software Diamond (E-value ≤ 1e−5) (Benjamin et al., 2015). According to the protein domains and motifs, the proteins were annotated against the InterPro and Pfam databases using InterProScan v5.36 software [56]. The annotation based on Gene Ontology (GO) terms also integrated the annotation results from InterPro.

Genome-wide prediction of ncRNAs was performed. MiRNAs, snRNAs and snoRNAs were annotated by comparison with the Rfam v14.1 library [57]. The tRNA sequences in the genome were annotated by tRNAscan-SE v1.3.1 software [58]. The model was constructed by Barrnap software [59] to predict rRNAs and their various subunits.

### Gene family and divergence time estimation

The genomes of ‘Lovell’ (v2.0), ‘Chinese Cling’, *Prunus apricot*, *Armeniaca mume* Sieb, and *Malus domestica* were downloaded from GDR (https://www.rosaceae.org/). The genomes of *Cucumis sativus*,* Solanum lycopersicum*, *Oryza sativa*, and* Arabidopsis thaliana* were downloaded from CuGenDB (http://www.cucurbitgenomics.org/), SGN (https://solgenomics.net/), NCBI (https://www.ncbi.nlm.nih.gov/), and TAIR (https://www.arabidopsis.org/). The similarity of protein sequences among the 10 species was obtained by all vs. all Blastp (v2.11.0+; e-value <10^-5^; https://www.blast.ncbi.nlm.nih.gov/Blast.cgi). The estimation of divergence time was performed with MCMCTree software (v4.9; parameters, clock = 3, model = 0; [60]). The expanded and contracted genes were analysed with the software Cafe5 (v5.0.0; [61]).

## Supplementary Information

**Additional file 1.**

**Additional file 2.**

## Data Availability

The raw sequencing data of the FCHL genome have been uploaded to NCBI, and the data numbers are SRR21053496-SRR21053498. The RNA-Seq data of FCHL flowers have been uploaded to NCBI, and the data numbers are SRR21227527- SRR21227538. The RNA-Seq data of FCHL leaves have been uploaded to NCBI, and the data numbers are SRR21155905-SRR21155913.

## Abbreviations

FCHL: Feichenghongli
FCBL: Fei Cheng Bai Li
LHSM: Longhuashuimi
RYP1: Rui You Pan 1
CN14: Zhongyoutao 14
LTR: long terminal repeat
SSRs: simple sequence repeats
TIRs: terminal inverted repeats
SVs: structural variants
USP: universal stress protein A-like protein
NLR: leucine-rich repeat
FTP1: the red dot period
FTP2: the budding flower period
FTP3: the full bloom period
DAM: dormancy-associated MADS-box
LDP1: the young leaf period
LDP2: the mature leaf period
