# Supplementary material for: A high-quality assembled genome of a representative peach landrace, ‘Feichenghongli’, and analysis of distinct late florescence and narrow leaf traits
Source: BMC Plant Biol. 2023 Apr 29;23:230. doi: 10.1186/s12870-023-04242-7 (PMC10148998; doi:10.1186/s12870-023-04242-7)
Supplement: Supplementary file 1 — Additional file 1. [file 12870_2023_4242_MOESM1_ESM.pdf]

Figure S1. Overview of the pipeline used for the FCHL genome assembly (picture drawn from Qingdao Ouyi Biotech Company, China).

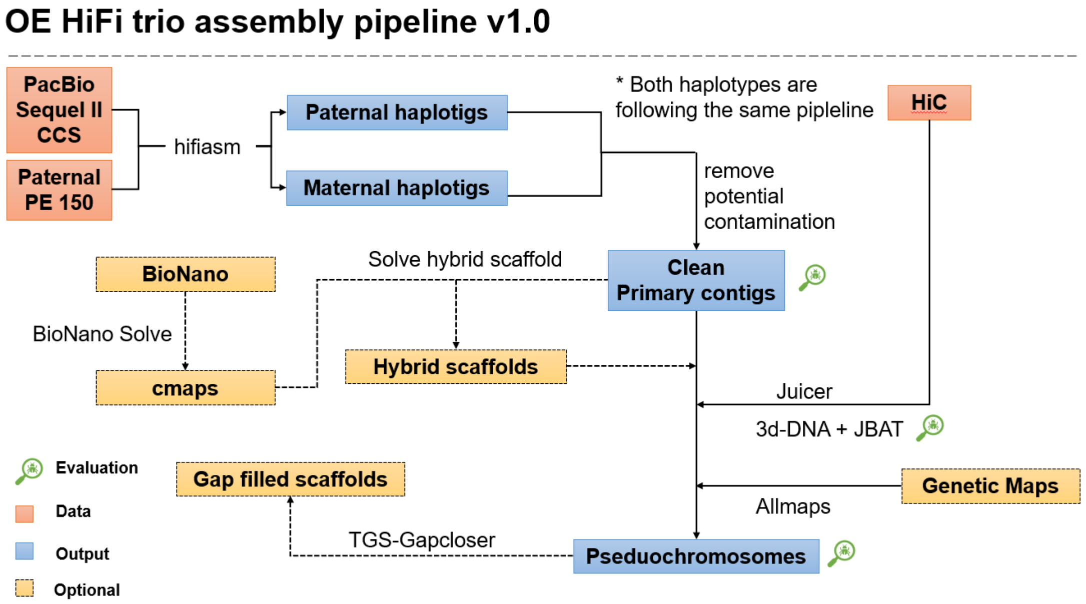

Figure S2. KAT distribution diagram of the FCHL genome at the contig assembly level

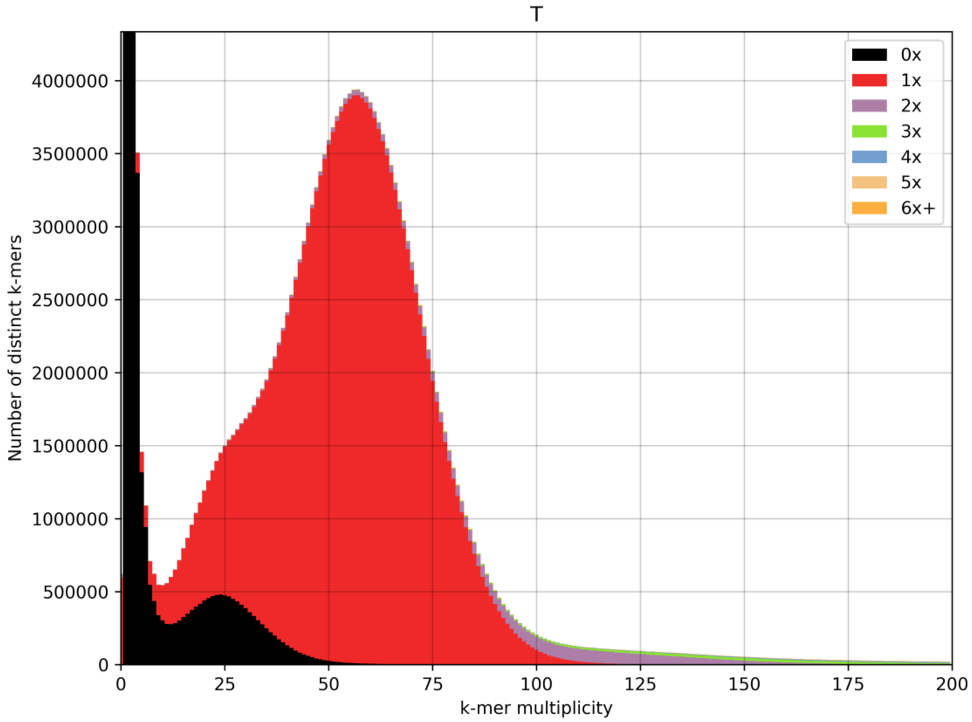

Figure S3. LAI chromosome distribution map of the FCHL genome

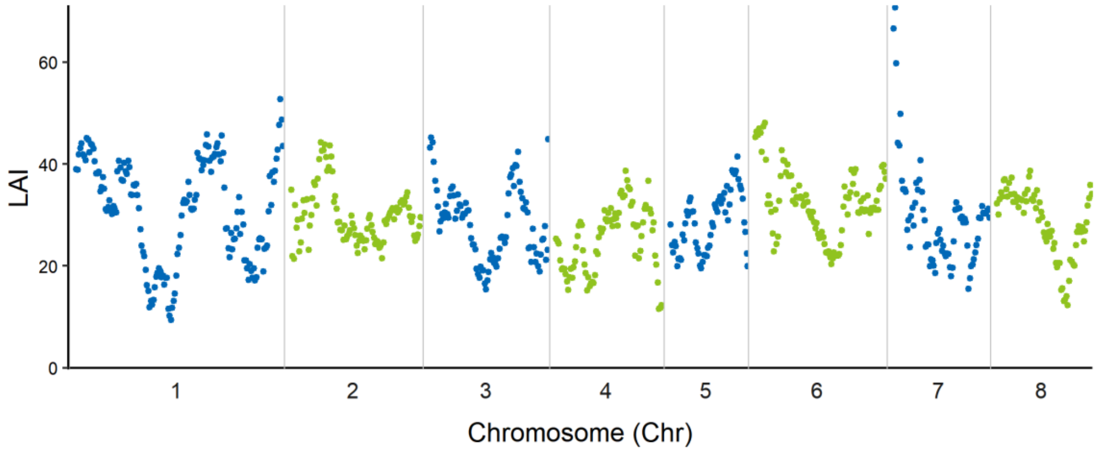

Figure S4. Estimation of insertion time of LTR transposons in the FCHL genome.

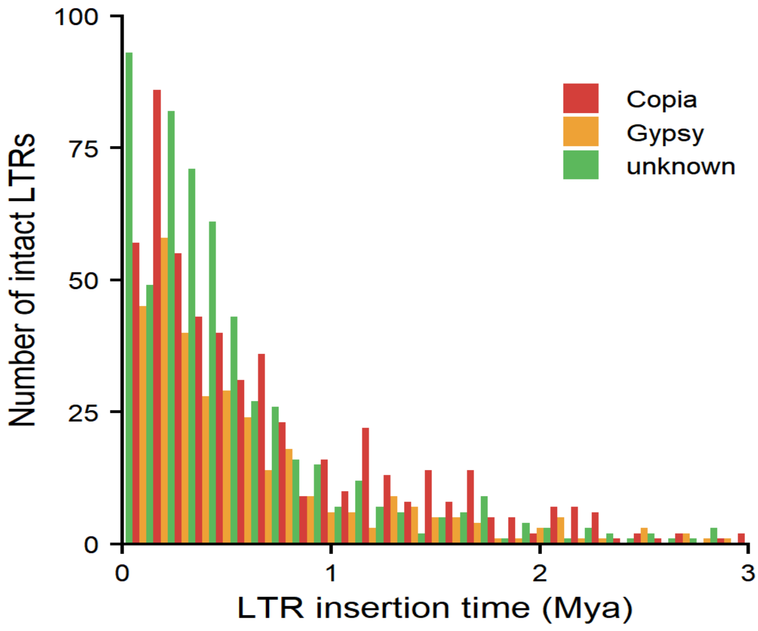

Figure S5. Distribution of LTR transposons of Copia type on the FCHL chromosomes

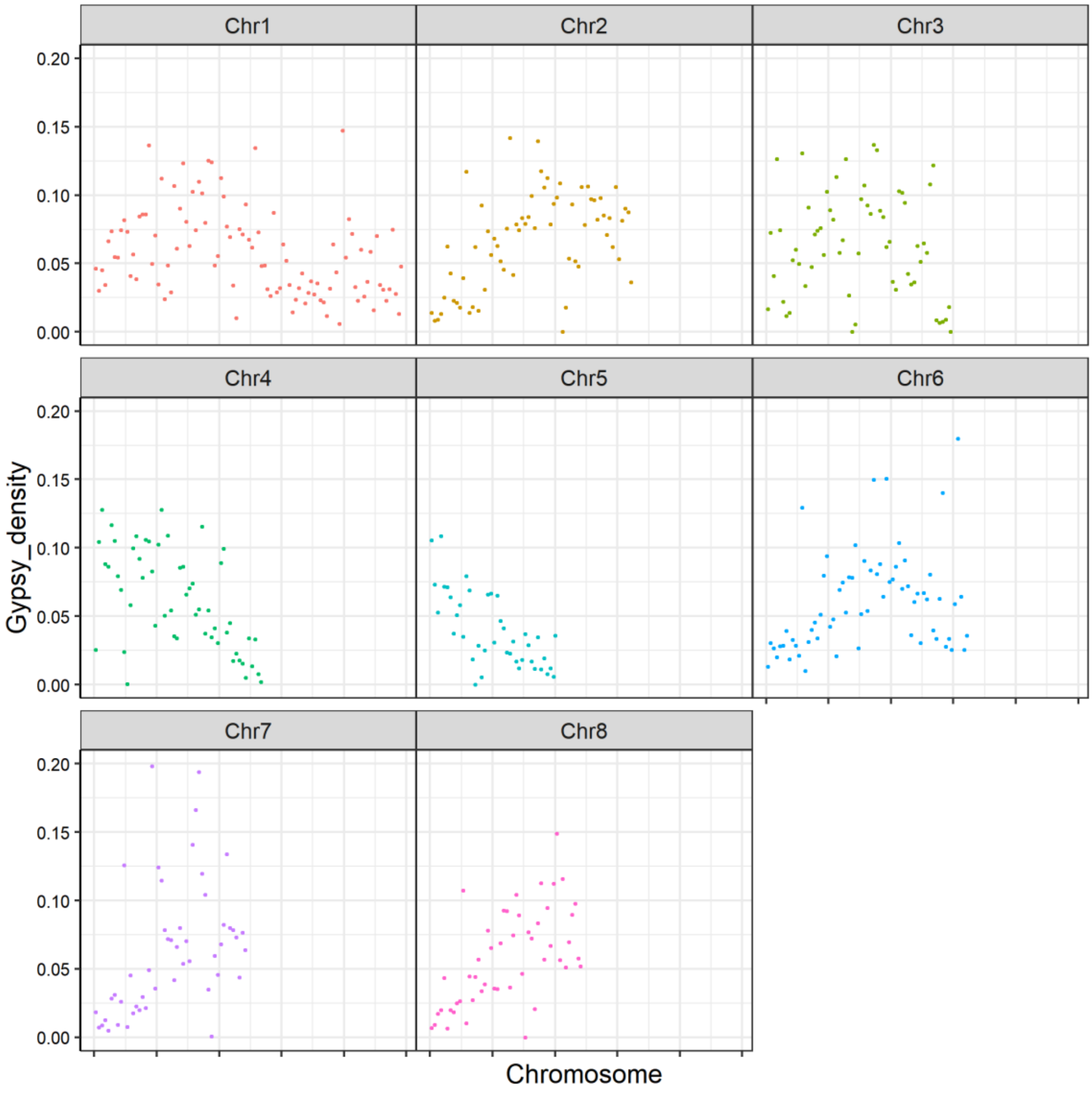

Figure S6. Distribution of Gypsy type LTR transposons on the FCHL chromosomes

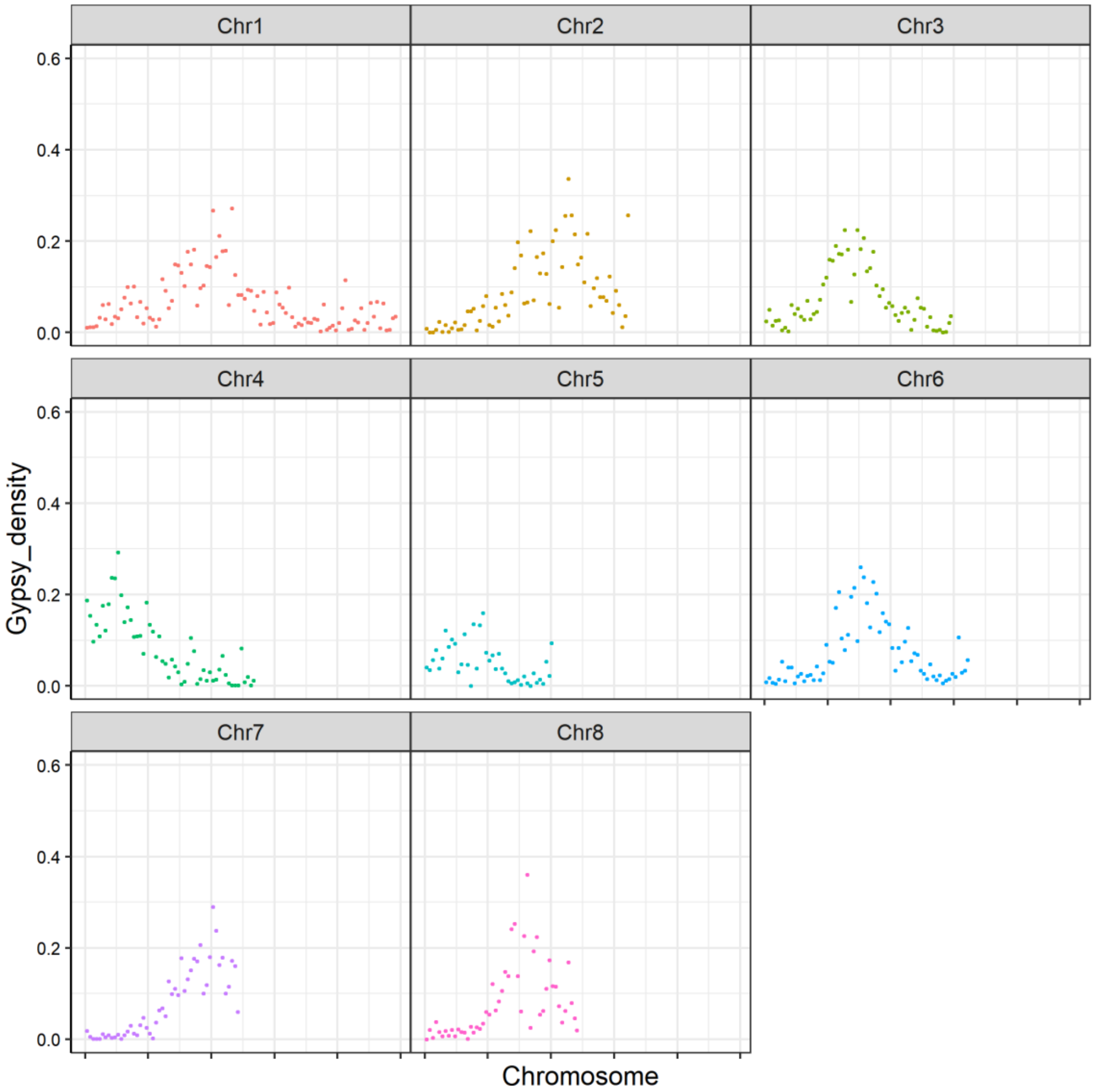

Figure S7. eggNOG Function Classification of the FCHL genes

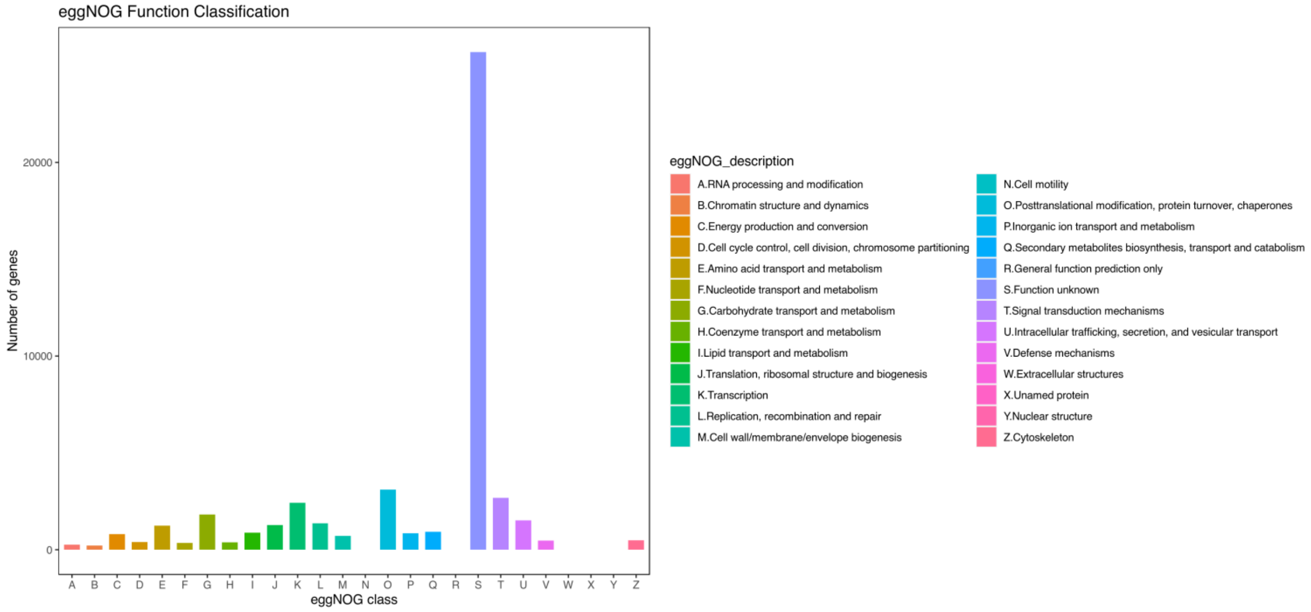

Figure S8. Gene ontology (GO) function classification of the FCHL genes

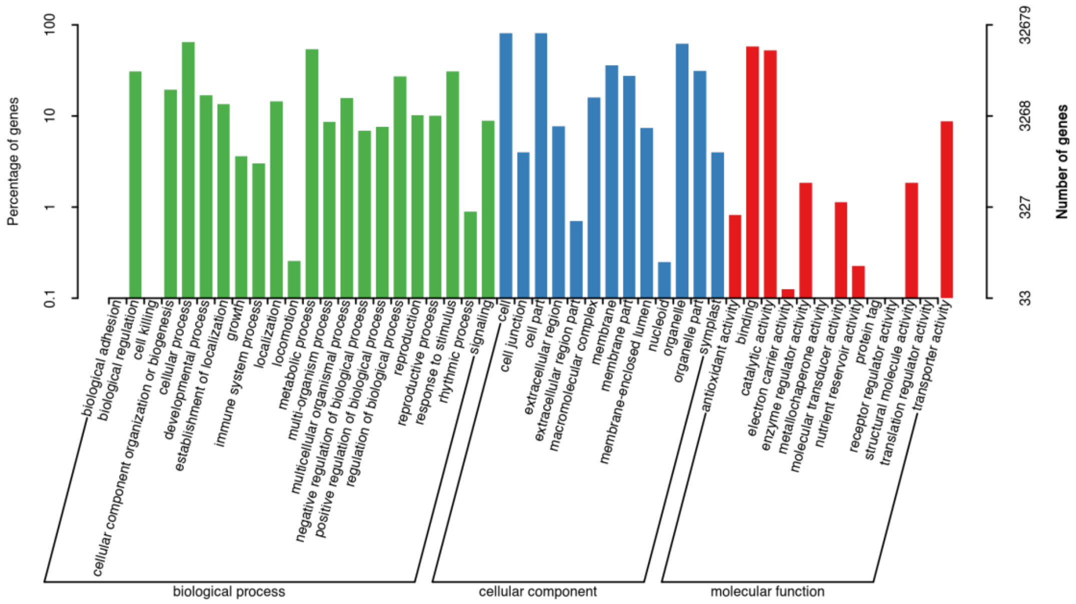

Figure S9. KEGG [1-3] classification of the FCHL genes

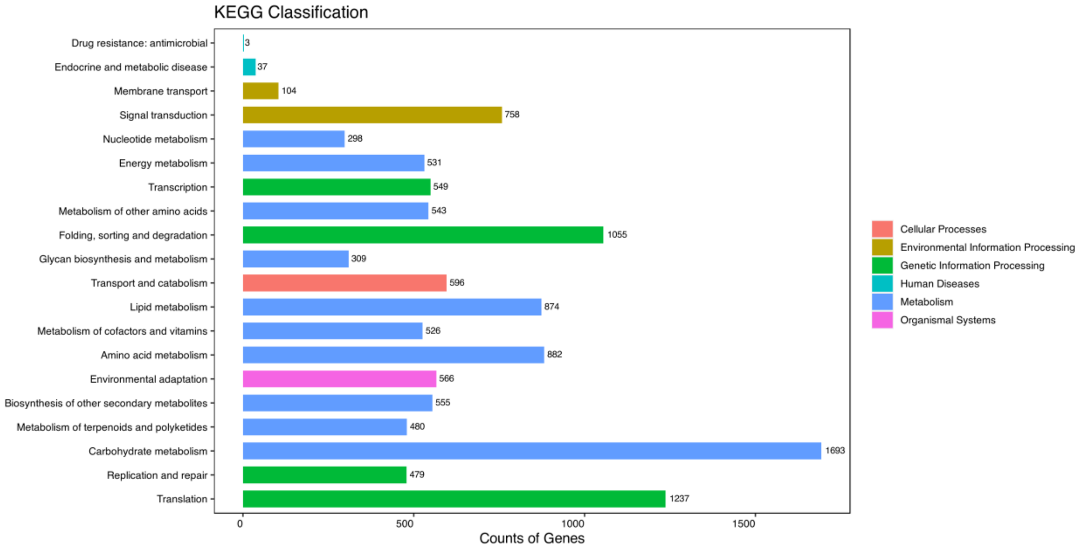

Figure S10. KOG classification of the FCHL genes

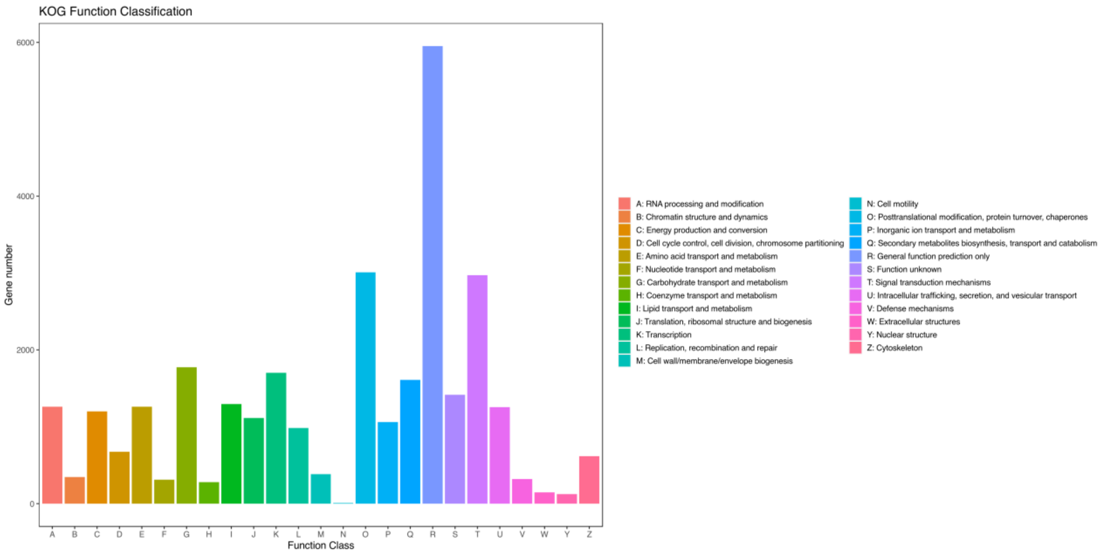

Figure S11. NR database analysis of the FCHL genes

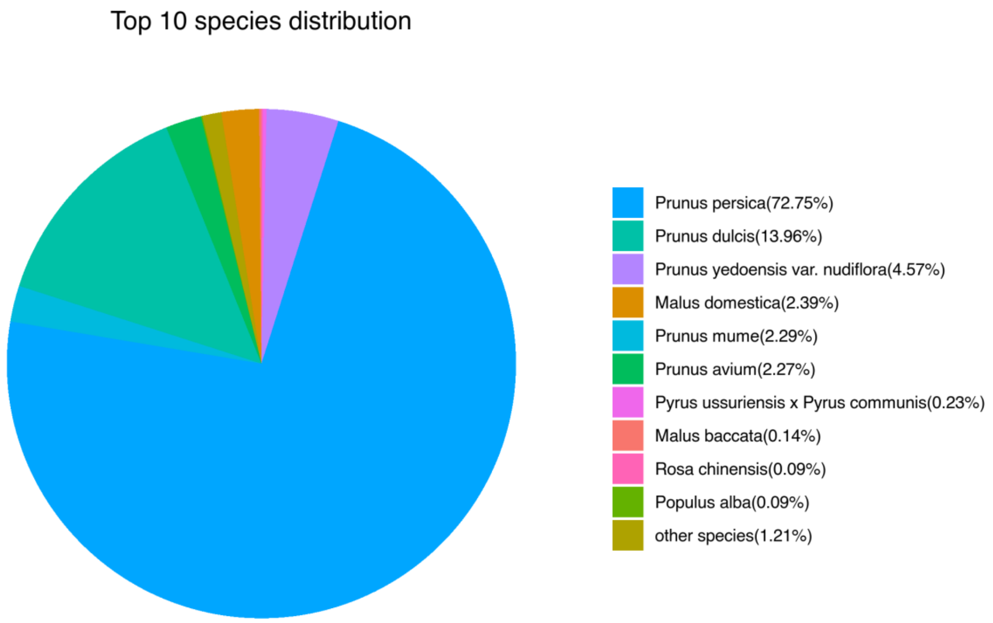

Figure S12. UpSet map of the FCHL genome in the nine databases

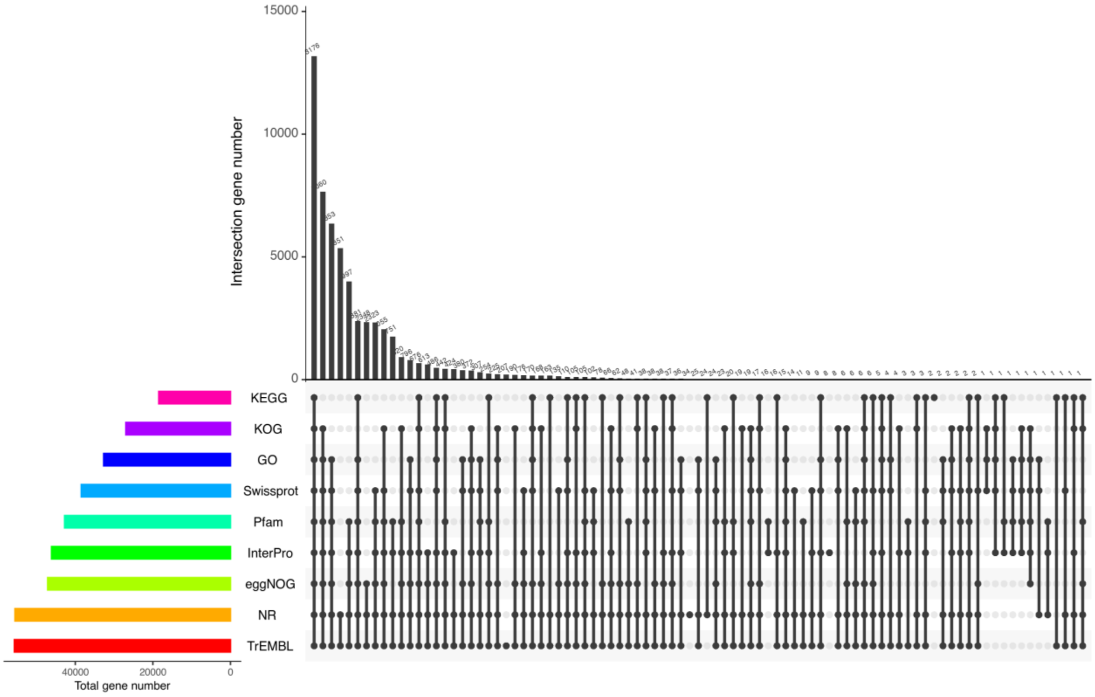

Figure S13. The KEGG [1-3] enrichment map of genes in the significantly contracted gene families in FCHL.

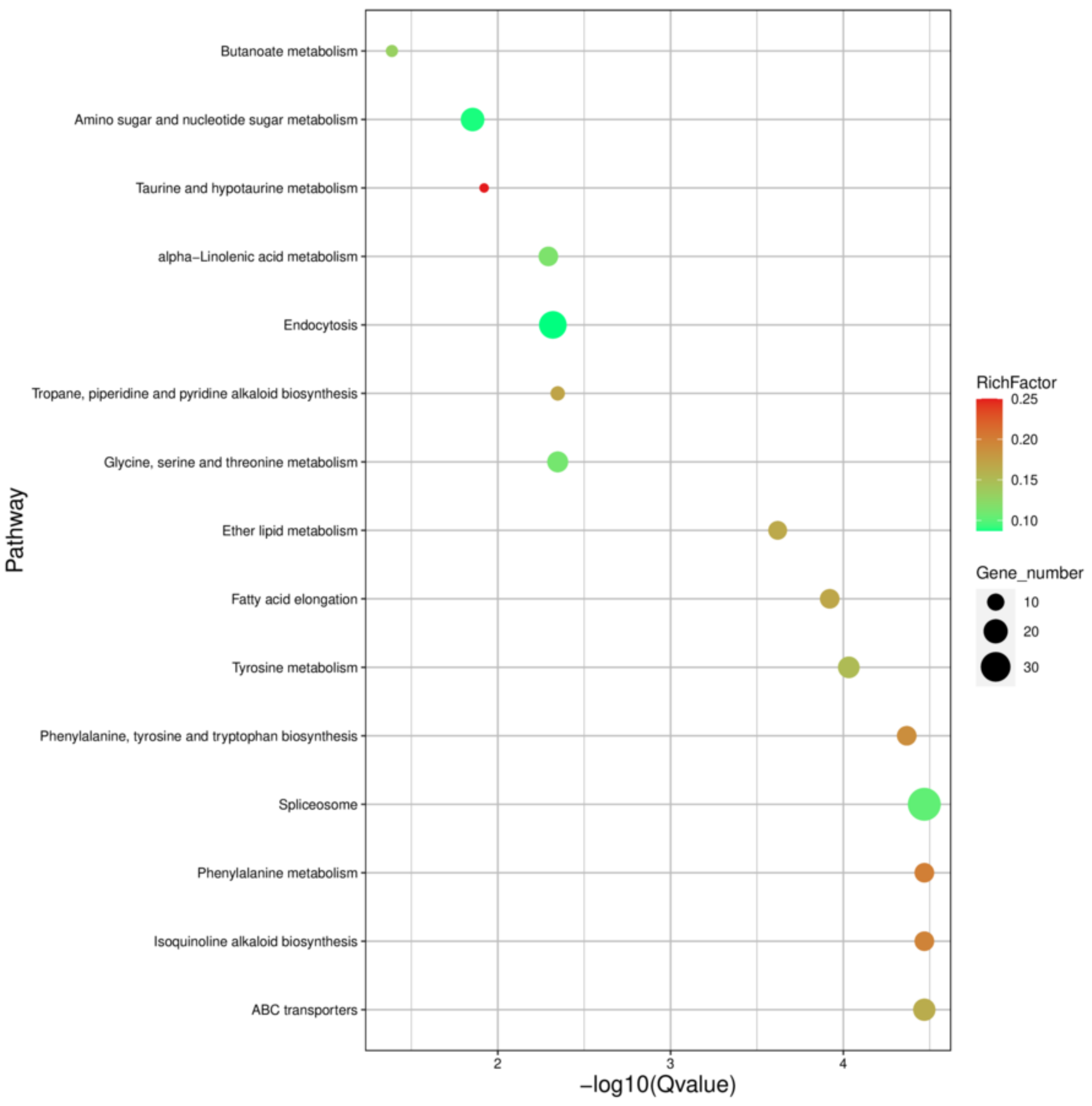

Figure S14. KEGG [1-3] enrichment map of genes in the significantly expanded gene families in FCHL.

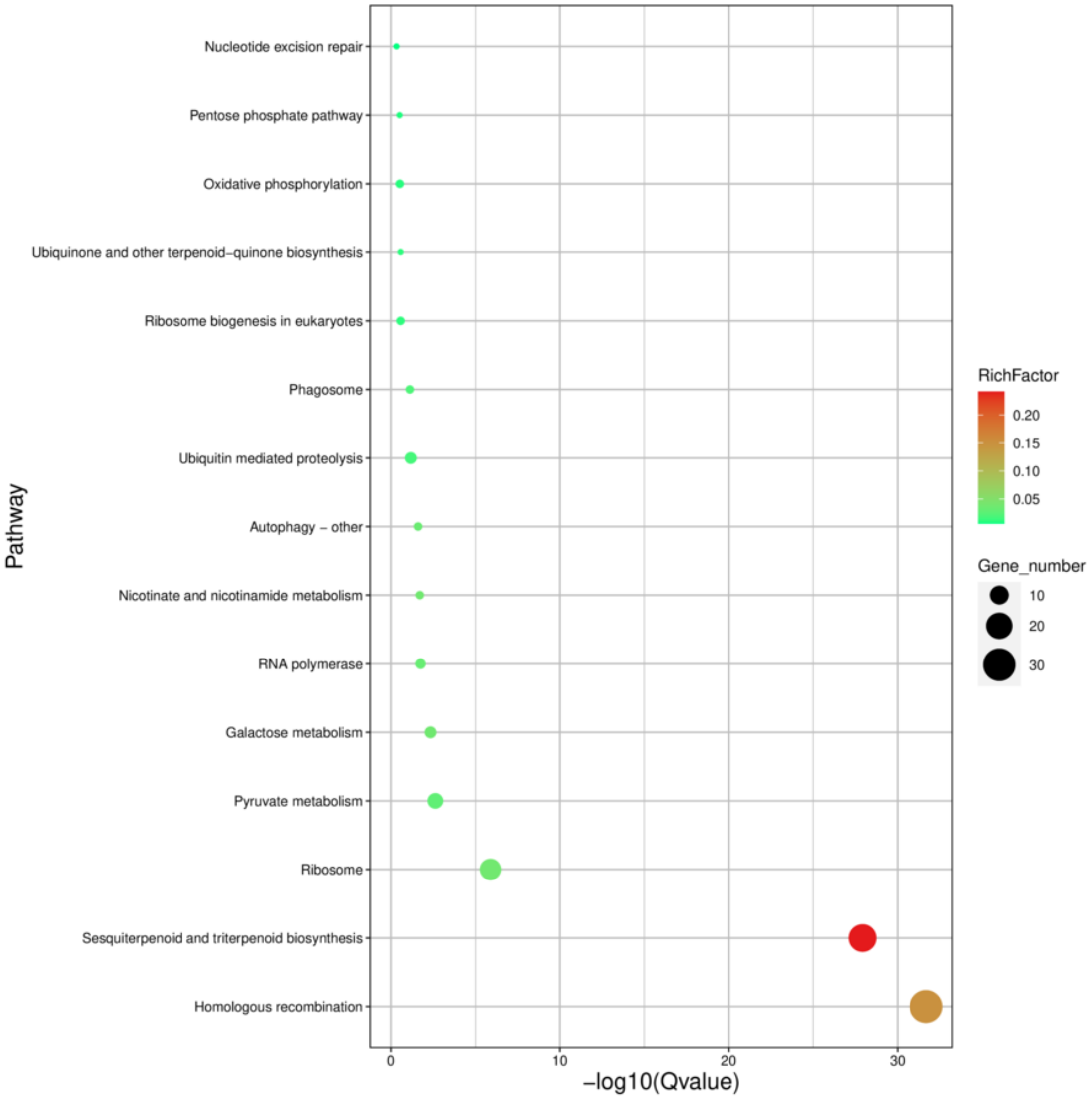

Figure S15. KEGG [1-3] enrichment analysis of FTP2 vs. FTP1.

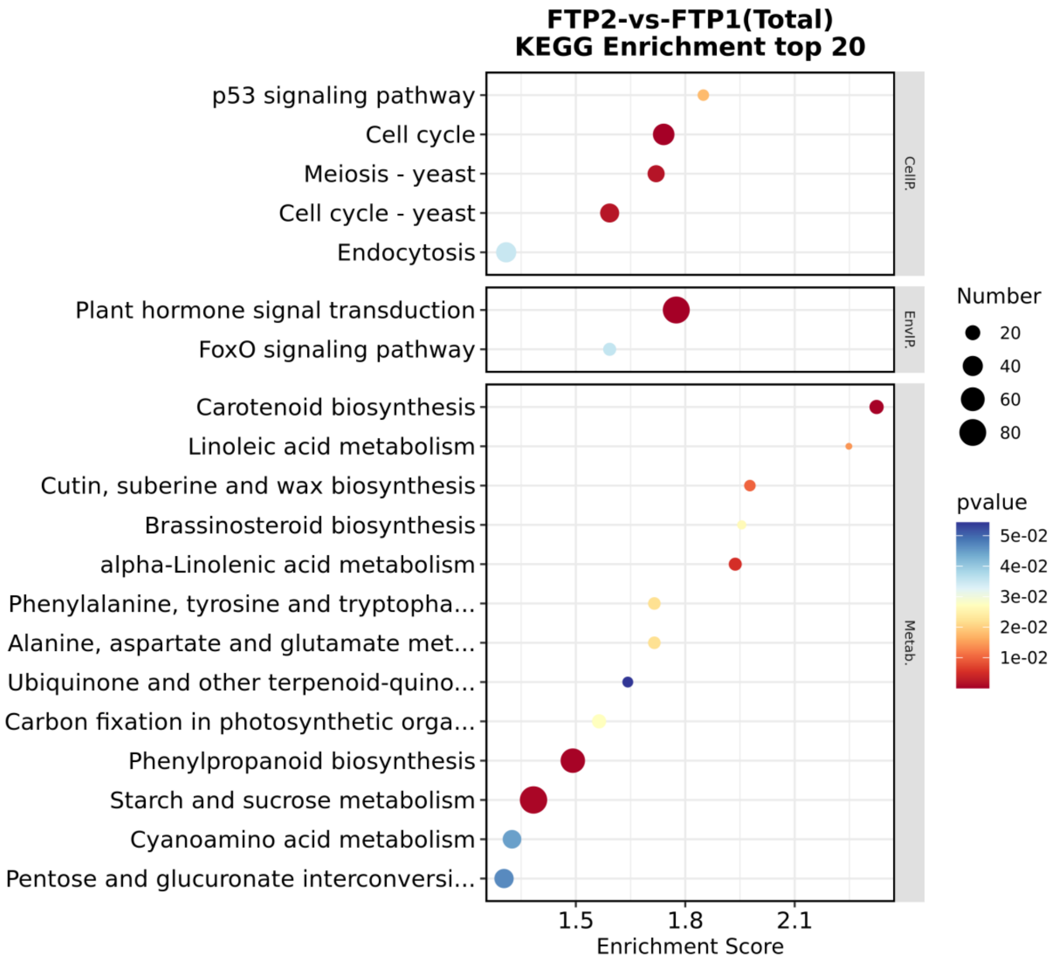

Figure S16. KEGG [1-3] enrichment analysis of FTP3 vs. FTP2

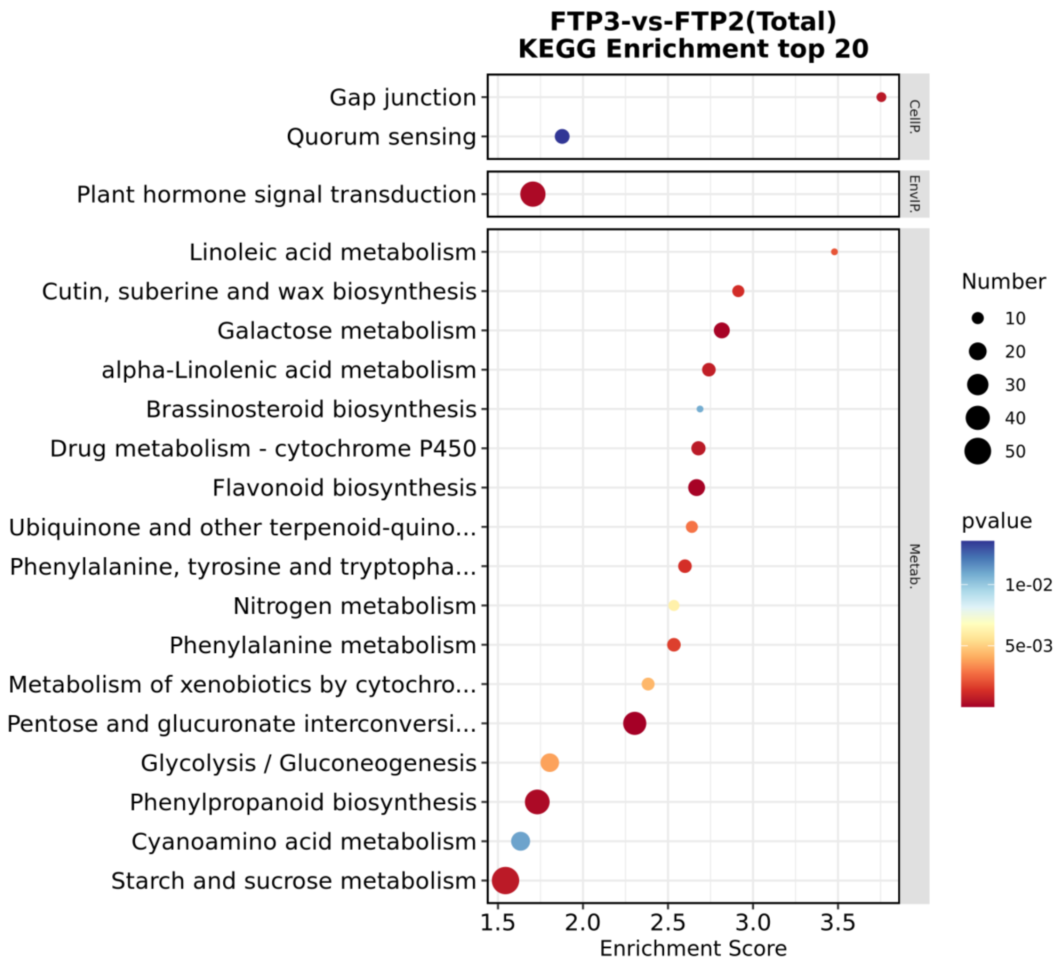

Figure S17. The fruit picture of ‘Feichenghongli ’

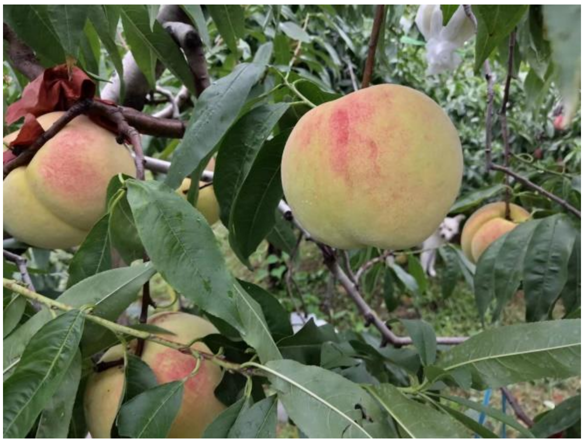

Figure S18. PCR confirmation of the variation in the FCHL gene Ppersica08G000108 (b) (The original, unprocessed version). M, marker; 1, FCHL; 2, Lovell; 3, the negative control; 4-5, other experiments not related to this experiment

Primer Information: FW: 5'- TAAAACTTGAACACTAACCATGTTCT -3'

RV: 5'- GTTTAGACTGTTTGTGGCATGAGTA -3'

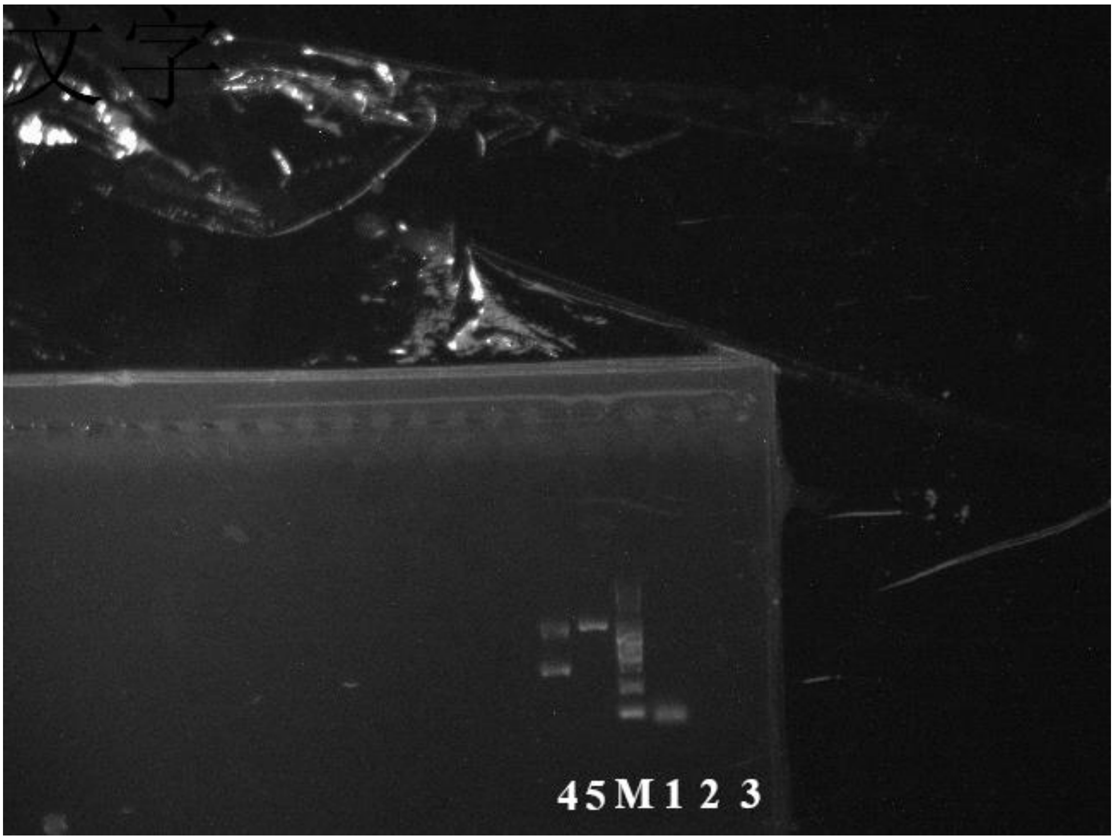

Reference

1.Kanehisa, M. and Goto, S.; KEGG: Kyoto Encyclopedia of Genes and Genomes. Nucleic Acids Res. 28, 27-30 (2000).

2.Kanehisa, M; Toward understanding the origin and evolution of cellular organisms. Protein Sci. 28, 1947-1951 (2019).

3.Kanehisa, M., Furumichi, M., Sato, Y., Kawashima, M. and Ishiguro-Watanabe, M.; KEGG for taxonomy-based analysis of pathways and genomes. Nucleic Acids Res. 51, D587-D592 (2023).
