# Supplementary material for: A high-quality assembled genome of a representative peach landrace, ‘Feichenghongli’, and analysis of distinct late florescence and narrow leaf traits
Source: BMC Plant Biol. 2023 Apr 29;23:230. doi: 10.1186/s12870-023-04242-7 (PMC10148998; doi:10.1186/s12870-023-04242-7)
Supplement: Supplementary file 2 — Additional file 2. [file 12870_2023_4242_MOESM2_ESM.pdf]

Table S1. Gene annotations on the FCHL 8 chromosomes

Chromsome	Length(bp)	gap_number	GC%	gene	protein
Chr1	49,322,632	0	37.42%	5948	12525
Chr2	32,331,349	0	37.62%	3622	7587
Chr3	29,510,740	1	37.44%	3392	7131
Chr4	26,930,668	0	37.57%	3117	6315
Chr5	20,138,513	1	38.22%	2454	4971
Chr6	32,278,396	1	37.5%	3980	7960
Chr7	24,323,596	0	37.51%	2800	5640
Chr8	24,231,253	1	37.9%	3332	6231
total	239.06	4	37.6%	28645	58360

Table S2. Repeat sequence statistics of the FCHL genome

Repeat	Class	Count	bpMasked	%masked
**LTR**	Copia	23621	13296156	5.56%
**LTR**	Gypsy	16833	16720278	6.99%
**LTR**	long_terminal_repeat	1029	112419	0.05%
**LTR**	unknown	21678	12200259	5.10%
**TIR**	CACTA	12249	6835800	2.86%
**TIR**	Mutator	74165	14581025	6.10%
**TIR**	PIF_Harbinger	15535	5746050	2.40%
**TIR**	Tc1_Mariner	1155	344042	0.14%
**TIR**	hAT	9939	3734343	1.56%
**nonTIR helitron**	helitron	23332	6540131	2.74%
**repeat_region**		1120	901272	0.38%
**total**	interspersed	200656	81011775	33.89%
**SSR**	Low_complexity	14730	724395	0.30%
**SSR**	Simple_repeat	81491	3254686	1.36%
**Total**		296877	84990856	35.55%

Table S3. Annotation rate statistics of the FCHL genome in various databases

Anno_Database	Annotated_Number	300<=length<1000	length>=1000
KEGG	18487(31.68 %)	11443(19.61 %)	1629(2.79 %)
KOG	26964(46.20 %)	17250(29.56 %)	2624(4.50 %)
GO	32679(56.00 %)	20578(35.26 %)	2910(4.99 %)
Swissprot	38452(65.89 %)	24496(41.97 %)	3601(6.17 %)
Pfam	42752(73.26 %)	26156(44.82 %)	3627(6.21 %)
InterPro	46117(79.02 %)	27469(47.07 %)	3837(6.57 %)
eggNOG	47112(80.73 %)	27791(47.62 %)	3934(6.74 %)
NR	55541(95.17 %)	28881(49.49 %)	3964(6.79 %)
TrEMBL	55692(95.43 %)	28882(49.49 %)	3964(6.79 %)
Total	55784(95.59 %)	28886(49.50 %)	3964(6.79 %)

Table S4. The RNA-seq data quality results of three duplications in three flower periods. FTP1,the red dot period; FTP2, budding flower period; FTP3, full bloom period

Sample	Phrase	RawReads	RawBases	CleanReads	CleanBases	ValidBases	Q30	GC
Flower1	FTP1	51.21M	7.68G	50.17M	7.41G	96.46%	92.65%	45.35%
Flower2	FTP1	45.75M	6.86G	44.76M	6.62G	96.52%	92.40%	45.58%
Flower3	FTP1	47.70M	7.15G	46.71M	6.91G	96.57%	92.49%	45.51%
Flower4	FTP2	46.83M	7.02G	45.80M	6.77G	96.31%	92.49%	46.21%
Flower5	FTP2	44.21M	6.63G	43.29M	6.40G	96.43%	92.59%	45.79%
Flower6	FTP2	45.15M	6.77G	44.21M	6.53G	96.43%	92.53%	45.74%
Flower7	FTP3	47.92M	7.19G	46.97M	6.94G	96.54%	92.68%	45.53%
Flower8	FTP3	45.30M	6.80G	44.31M	6.55G	96.37%	92.32%	45.85%
Flower9	FTP3	47.07M	7.06G	45.87M	6.77G	95.87%	91.53%	45.61%

Table S5. The RNA-seq data quality results of three duplications in two leaf development periods of the FCHL and Zhongyou 4. LDP1,young leaf period; LDP2,mature leaf period; **FL, FCHL Leaf; ZL, Zhongyou 4 Leaf.**

Sample	Phrase	RawReads	RawBases	CleanReads	CleanBases	ValidBases	Q30	GC
FL1_1	LDP1	48.65M	7.30G	47.60M	7.06G	96.74%	93.17%	45.32%
FL1_2	LDP1	50.22M	7.53G	49.02M	7.27G	96.45%	92.58%	45.36%
FL1_3	LDP1	46.12M	6.92G	45.43M	6.73G	97.22%	94.14%	45.57%
FL2_1	LDP2	45.71M	6.86G	44.90M	6.66G	97.14%	93.54%	45.29%
FL2_2	LDP2	46.69M	7.00G	45.98M	6.83G	97.52%	93.49%	44.53%
FL2_3	LDP2	47.08M	7.06G	46.26M	6.86G	97.19%	93.57%	45.51%
ZL1_1	LDP1	45.53M	6.83G	44.90M	6.68G	97.78%	94.52%	44.85%
ZL1_2	LDP1	49.49M	7.42G	48.89M	7.28G	97.99%	95.10%	44.67%
ZL1_3	LDP1	43.47M	6.52G	42.87M	6.37G	97.77%	94.67%	44.99%
ZL2_1	LDP2	47.35M	7.10G	46.65M	6.92G	97.38%	94.34%	45.39%
ZL2_2	LDP2	45.19M	6.78G	44.54M	6.60G	97.44%	94.26%	45.45%
ZL2_3	LDP2	46.07M	6.91G	45.35M	6.72G	97.25%	93.98%	45.62%

TableS6 Statistics of mesophyll cells

	FCHL	Zhongyou 4
Average layers of mesophyll cells	6	6
Length of mesophyll cells of upper epidermis (um)	20.66±0.42	20.46±0.81
Length of mesophyll cells of lower epidermis (um)	15.94±0.74	15.87±0.69
Number of mesophyll cells/0.01mm^2	24.6	30.6*

Values are means±SD. T The statistical significance of differences between FCHL and Zhongyou 4 was analyzed using a t-test: *P < 0.05
